# Relationship Between Predicted Habitat Suitability and Population Density of Game Mammals Under a Sharply Continental Climate

**DOI:** 10.1002/ece3.74621

**Published:** 2026-11-08

**Authors:** Zarina Sergazinova

**Affiliations:** ^1^ Department of Biology and Ecology, Faculty of Natural Science Toraighyrov University NCJSC Pavlodar Kazakhstan

**Keywords:** biodiversity, climatic factors, ecological niche, habitat suitability, mammals, MaxEnt, population density, species distribution models

## Abstract

This study evaluates the relationship between MaxEnt‐predicted habitat suitability and departmental estimates of population density for 14 game mammal species in the Pavlodar Region. Model performance varied substantially among species (Test AUC = 0.538–0.992; TSS = 0.220–0.984), and extremely high AUC values for some species with small samples were interpreted cautiously. For 41 territorial units, habitat suitability was aggregated as the mean MaxEnt value within each polygon and compared with density using Spearman's rank correlation. Spearman's *r*_s_ ranged from 0.288 to 0.738; in the initial analysis, a statistically significant positive relationship was detected for 13 of the 14 species, whereas the relationship was not statistically significant for the steppe polecat (*r*_s_ = 0.288; *p* = 0.068). Residual spatial autocorrelation assessed with Moran's *I* was non‐significant for 11 species; for wolf, bobak marmot, and roe deer, an additional spatially constrained permutation analysis was performed. After this analysis, the relationship remained significant for wolf (*r*_s_ = 0.714, *p* = 0.0007) and roe deer (*r*_s_ = 0.488, *p* = 0.0198), whereas for bobak marmot it remained positive but was not statistically significant after accounting for spatial structure (*r*_s_ = 0.467; *p* = 0.0615). The results indicate that habitat suitability reflects the environmental potential of an area but is not a direct measure of population density.

## Graphical abstract

Understanding where animals live and what their population sizes are is crucial for nature conservation and the sustainable use of natural resources. Computer models are widely used in modern ecological research to predict suitable habitats for species; however, such predictions do not always reflect the actual structure of populations. This study analyzed 15 mammal species in the harshly continental climate of north‐eastern Kazakhstan, and the results of habitat suitability modeling were compared with actual population density data collected in the field. It was shown that for species with narrow ecological specialization, for example, those associated with forest ecosystems, the predicted suitable areas generally coincide with zones of high abundance. At the same time, for ecologically plastic species capable of inhabiting various conditions, this relationship is significantly weaker. This indicates that habitat suitability alone does not always determine animal abundance. It has been established that climatic factors, particularly temperature conditions characteristic of a sharply continental climate, play an important role in shaping species ranges; however, local factors, such as food availability and anthropogenic impact, also exert a significant influence. The results obtained show that combining modeling approaches with field observations allows for a more accurate assessment of animal distribution and can be used to improve conservation measures, manage hunting resources, and monitor ecosystem changes.

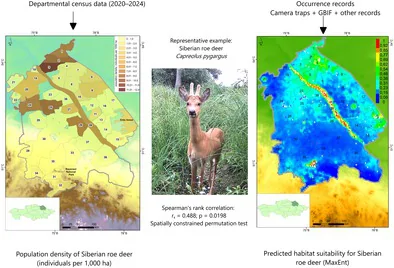

## 1 Introduction

Understanding the relationship between the potential suitability of habitats and actual population densities remains one of the key challenges in modern spatial ecology. Species distribution models (SDMs), particularly the maximum entropy (MaxEnt) algorithm, are widely used to estimate the potential range of species based on presence data. Nevertheless, it remains a matter of debate to what extent habitat suitability indices can be considered reliable indicators of actual population parameters, including abundance and density (Elith and Leathwick 2009; Merow et al. 2013).

A number of studies have shown that SDMs primarily describe a species' fundamental ecological niche, whereas realized population distribution and density are shaped by a broader set of factors, including biotic interactions, dispersal constraints, anthropogenic pressure, and the spatial structure of the food supply (Elith and Leathwick 2009; Merow et al. 2013). Consequently, the relationship between habitat suitability and population density is ambiguous and varies significantly among species.

Despite the active development of species distribution modeling (SDM) methods (Phillips et al. 2006; Elith et al. 2011), studies integrating predicted habitat suitability with actual population density data remain limited, especially for complexes of game mammals that differ in their degree of ecological specialization and mobility.

Under a sharply continental climate, temperature seasonality and annual temperature range may act as important ecological filters; however, the expected strength of the relationship between habitat suitability and observed density depends not only on ecological specialization but also on resource availability, anthropogenic pressure, mobility, detectability, and the spatial scale of analysis. Therefore, differences between specialized and ecologically plastic species are treated here as a testable hypothesis rather than as a predetermined ecological rule.

This issue is particularly relevant for regions with a sharply continental climate, such as northeastern Kazakhstan, where pronounced climatic gradients, high seasonal temperature ranges, and significant landscape heterogeneity shape the complex spatial structure of faunal complexes.

Contemporary studies of the region's mammalian fauna are predominantly fragmentary and sporadic in nature. The fundamental basis for the study of Kazakhstan's mammals was laid in the seminal works of Belov (1931), Kuznetsov (1948), Afanasyev et al. (1953), Shnitnikov (1935), as well as in the multi‐volume publication «Mammals of Kazakhstan» edited by Sludsky (1985, 1977, 1978). Subsequent studies of the regional fauna, including the Pavlodar Pre‐Irtysh region, were further developed in the works of Bazarbekov and Lyakhov (2005), Litvinov et al. (2015), Erzhanov et al. (2016), as well as in later publications (Dupal et al. 2017; Sergazinova, Makarov, Toleuzhanova, et al. 2025; Sergazinova, Makarov, Erzhanov, et al. 2025).

This study employs an integrative approach that combines species distribution modeling (MaxEnt) with independent data on population density (departmental data based on winter route surveys). Unlike most studies focused on assessing the potential suitability of habitats, this study compares the potential and actual distribution of species. This approach allows us to assess the spatial organization of populations and identify discrepancies between the ecological suitability of the environment and the actual distribution of species. The study was conducted in the poorly studied region of northeastern Kazakhstan, which enhances its scientific and applied significance.

The objective of this study is to assess the correlation between the predicted habitat suitability and the actual population density of 14 mammal species in the Pavlodar Region, while identifying the factors that determine the differences between the potential and actual distribution of species.

To achieve this objective, the following hypotheses were formulated:

Hypothesis 1 *There is a positive relationship between habitat suitability and population density, but its strength varies among species*.

Hypothesis 2 *Species with narrow ecological specialization demonstrate greater consistency between suitability and density compared to generalists*.

Hypothesis 3 *Steppe and ecologically plastic species are characterized by a more pronounced mismatch between habitat suitability and actual population density*.

## 2 Materials and Methods

The research was conducted in the Pavlodar Region, located in the far northeast of the Republic of Kazakhstan in the forest‐steppe, steppe, and semi‐desert zones along the middle reaches of the Irtysh River. The Pavlodar Region is situated in the central part of the Eurasian continent, almost equidistant from the Atlantic and Pacific Oceans. The geographical coordinates of the region's extreme points are 49°55′–54°27′ north latitude and 73°16′–79°56′ east longitude. The climate is sharply continental (Insebayev et al. 2007). The Pavlodar Region covers an area of 127,500 km^2^. In the north, the region borders the Omsk Region; in the northeast, the Novosibirsk Region; and in the east, the Altai region of the Russian Federation; in the south, the Abai and Karaganda regions; and in the west, the Akmola and North Kazakhstan regions of the Republic of Kazakhstan. The administrative center of the region is the city of Pavlodar (Aryn 2003).

The territory of Pavlodar Region is rich in natural resources, unique ecosystems, and diverse flora and fauna. Hunting reserves, reserve fund hunting grounds, and specially protected natural areas contribute to the conservation of biological diversity, ensuring the sustainable use of natural resources and the protection of ecosystems. In total, there are 32 hunting reserves and 6 reserve fund hunting grounds in the region (Figure 1) (Pavlodar Society of Hunters and Fishermen, n.d.). In addition, the region contains specially protected natural areas (SPNAs): the Republican State Institution «Ertis Ormany State Forest Nature Reserve» (hereinafter referred to as the RSI SFNR «Ertis Ormany»), the Republican State Institution Bayanaul State National Nature Park (hereinafter referred to as the RSI BSNNP), the state nature reserves «Irtysh River Floodplain» (comprehensive) and «Kyzyltau» (zoological), as well as the State Natural Monument «Goose Migration», which is of significant scientific interest as a paleontological site.

**FIGURE 1:**
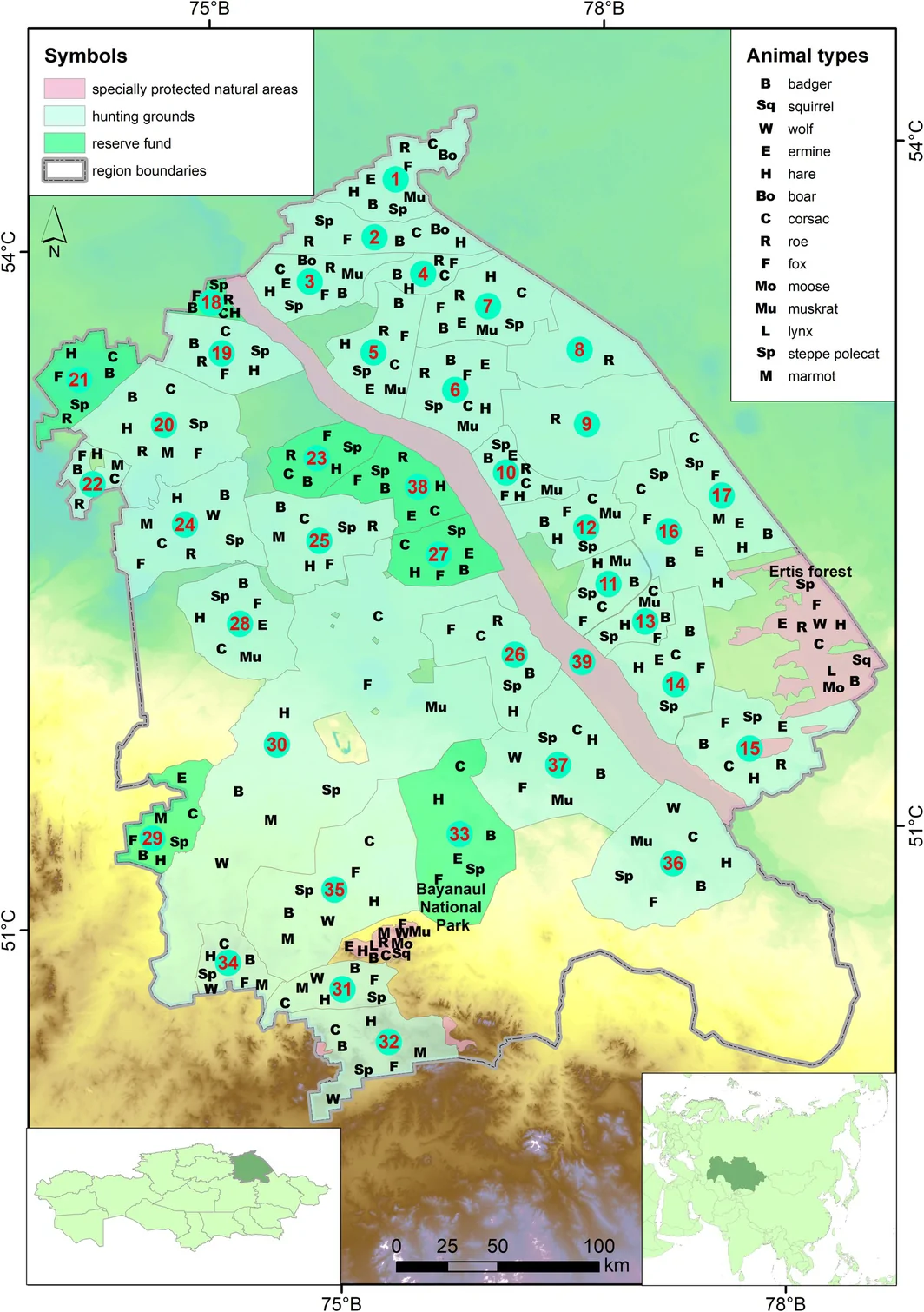
Spatial distribution of habitats of game mammals in the Pavlodar Region.

Datasets with independent analytical purposes were used. Departmental data provided by authorized organizations on the abundance of 14 game mammal species in the Pavlodar Region for 2020–2024 (Table 1) were used to calculate population density. The resulting density estimates were subsequently used for an independent comparison with predicted habitat suitability. Presence data used to build the MaxEnt models were compiled separately and included the author's field observations, departmental occurrence records, questionnaire survey data, and records from the international open‐access GBIF database (GBIF.org 2026a, 2026b, 2026c, 2026d, 2026e, 2026f, 2026g, 2026h, 2026i, 2026j, 2026k, 2026l, 2026m, 2026n). Records were obtained not only from the Pavlodar Region but also from adjacent areas with similar environmental and climatic conditions, mainly the Novosibirsk, Omsk, and Tomsk regions, partly the Kemerovo Region (Russia), and northeastern Kazakhstan. Before modeling, records were checked for geographic coordinates and taxonomic identity; duplicate records and records with obviously erroneous or invalid coordinates were excluded. No additional spatial rarefaction or thinning of occurrence records was performed. After matching the records to the spatial extent of the environmental rasters, 699 presence points were used in the final models, of which 697 (99.71%) were located within the Pavlodar Region. For 12 species, all points used were within the region; for *Canis lupus* , 38 of 39 points were in the Pavlodar Region, and for *Vulpes corsac* , 24 of 25. One point for each of these species was located in the neighboring Karaganda Region. Thus, despite the broader geographic coverage of the original downloads from open databases, the final models were based almost entirely on regional data. Because the modeling and subsequent analyses were focused primarily on the Pavlodar Region, a separate MESS analysis was not performed.

**TABLE 1:** Population density of commercial mammals for the period 2020–2024, specimens/1000 ha.

No.	Land use category animal species	Fur‐bearing species											Ungulate species		
		W—wolf	L—lynx	F—fox	C—corsac	E—ermine	Sp—steppe polecat	B—badger	M—marmot	Mu—muscrat	H—hare	Sq—squirrel	Rd—roe deer	Мo—moose	Bo—wild boar
Hunting reserves															
1	Ozernoye (Zhelezin District)	0	0	1.08	0.58	0.86	0.75	0.44	0	7.16	4.52	0	0.93	0	0.06
2	Zelenaya Roscha (Zhelezin District)	0	0	0.83	0.24	0.73	0.59	0.34	0	6.53	5.82	0	0.69	0	0.04
3	Zakharovskoye (Zhelezin District)	0	0	1.12	0.40	0.76	0.73	0.57	0	21.39	5.62	0	0.64	0	0.05
4	Berezovskoye (Zhelezin District)	0	0	2.96	1.02	0	0	0.75	0	0	3.82	0	0.35	0	0
5	Kachirskoye (Kachir District)	0	0	0.70	0.55	0.25	0.74	0.65	0	2.31	1.75	0	0.27	0	0
6	Lvovskoye (Kachir District)	0	0	0.77	0.55	0.27	0.84	0.83	0	2.69	2.83	0	0.37	0	0
7	Trofimovskoye (Kachirsky District)	0	0	0.78	0.52	0.20	0.94	0.85	0	5.38	3.06	0	0.36	0	0
8	Lozovskoye (Uspensky District)	0	0	0	0	0	0	0	0	0	0	0	0.56	0	0
9	Pavlovskoye (Uspensky District)	0	0	0	0	0	0	0	0	0	0	0	0.29	0	0
10	Chernorechnoe (Pavlodar District)	0	0	1.92	0.80	0.04	1.08	2.61	0	4.37	4.04	0	0.47	0	0
11	Maraldy (Pavlodar District)	0	0	0.87	0.54	0	0.89	0.92	0	0.72	8.10	0	0	0	0
12	Zangar (Pavlodar District)	0	0	1.01	0.36	0	1.58	0.62	0	0.53	5.08	0	0	0	0
13	Olginka (Pavlodar District)	0	0	1.04	0.61	0	1.30	0.59	0	1.35	10.71	0	0	0	0
14	Yamyshevo (Lebyazhinsky District)	0	0	0.92	0.68	0.12	1.13	0.52	0	0	2.68	0	0	0	0
15	Lebyazhye (Lebyazhinsky District)	0	0	0.91	0.90	0.21	0.78	0.66	0	0	2.79	0	0.07	0	0
16	Maryanovka (Shcherbaktinsky District)	0	0	1.33	1.67	0.59	1.17	0.80	0	0	4.51	0	0	0	0
17	Shcherbakty (Shcherbaktinsky District)	0.02	0	2.34	3.64	1.19	3.74	1.03	12.55	0	8.92	0	0	0	0
19	Irtyshskoye (Irtyshsky District)	0	0	0.89	0.62	0	0.70	0.79	0	0	2.97	0	0.49	0	0
20	Panfilovskoye (Irtyshsky District)	0	0	0.40	0.20	0	0.37	0.43	0	2.31	2.48	0	0.29	0	0
22	Seleta (Irtyshsky District)	0	0	1.81	0.69	0	1.45	1.74	100.07	0	6.14	0	0.56	0	0
24	Ivanovskoye (Aktogay District)	0.01	0	0.43	0.18	0	0.55	0.22	5.67	0	2.43	0	0.43	0	0
25	Razumovskoye (Aktogay District)	0	0	0.64	0.28	0	0.54	0.24	7.31	0	2.88	0	0.44	0	0
26	Aksu (Aksu City Administration)	0	0	1.20	0.41	0	0.87	0.47	0	0	4.51	0	0.85	0	0
28	Zelenaya Roscha (Ekibastuz City Administration)	0	0	0.85	0.33	0	0.71	0.39	0	0	4.42	0	0	0	0
29	Bozshakol (Ekibastuz City Administration)	0	0	1.28	1.75	1.38	1.67	0.31	21.64	0	4.26	0	0	0	0
30	Ekibastuzskoye (Ekibastuz City Administration)	0.02	0	0.70	0.28	0	0.48	0.09	4.67	0.55	1.81	0	0	0	0
31	Bayanaulskoye (Bayan‐Aul District)	0.16	0	0.99	0.43	0	0.63	0.41	19.18	0	5.45	0	0	0	0
32	Yuzhnoye (Bayan‐Aul District)	0.07	0	0.78	0.34	0	0.73	0.27	129.94	0	2.49	0	0	0	0
34	Uzynbulak (Bayan‐Aul District)	0.13	0	1.24	0.51	0	0.85	0.44	124.27	0	5.12	0	0	0	0
35	Zhana‐Zhol (Bayan‐Aul District)	0.04	0	0.54	0.22	0	0.34	0.11	30.00	0	1.62	0	0	0	0
36	Mayskoye (Maysky District)	0.03	0	0.54	0.32	0	0.51	0.19	0	1.03	1.75	0	0	0	0
37	Bestau (Maysky District)	0.01	0	0.54	0.27	0	0.86	0.22	0	0.73	1.68	0	0	0	0
Reserve fund															
18	Zharbulak (Irtysh District)	0	0	3.04	3.18	0	5.22	2.47	0	0	12.67	0	0.92	0	0
21	Kyzylkak (Irtysh District)	0	0	0.31	0.41	0	0.48	0.30	0	0	1.27	0	0.23	0	0
23	Dzambulskoye (Aktogay District)	0	0	1.29	1.90	0	1.69	0.63	0	0	4.23	0	0.61	0	0
27	Kyzylzhar (Aksu City Administration)	0	0	1.63	2.05	1.34	1.44	0.47	0	0	7.00	0	0	0	0
33	Maikainskoye (Bayan‐Aul District)	0.02	0	0.67	1.14	0.79	2.10	0.21	0	0	1.20	0	0	0	0
38	Irtyshskoye (Aktogay District)	0	0	1.14	1.36	0.66	1.50	0.58	0	0	4.24	0	0.54	0	0
SPNA															
	RSI SFNR «Ertis Ormany»	0.15	0.14	1.43	0.65	0.34	1.34	1.43	0	0	5.59	22.01	2.60	0.62	0
	RSI BSNNP	1.17	0.50	2.54	1.08	0.69	0	2.22	177.29	1.94	57.97	23.01	1.26	0.34	0
39	Irtysh river floodplain	0	0	3.67	2.23	0.46	1.32	0.85	0	5.07	13.97	0	4.52	0	0.08

*Note:* Territorial‐unit numbers correspond to the codes in Figure 1; therefore, the row numbering in Table 1 is not sequential.

Occurrence data obtained from GBIF had species‐specific temporal coverage and included both historical and contemporary records. The overall temporal range of the original GBIF data was 1847–2025: the earliest records were for *Vulpes corsac* (1847), whereas for several species the time series began only in the 2000s. The temporal mismatch between occurrence data and environmental predictors was not statistically corrected and was treated as a limitation when interpreting the modeling results.

Field surveys were conducted in 2025 and included camera‐trap monitoring. Motion‐triggered camera traps were deployed from July to October 2025 in forest and floodplain ecosystems of the Pavlodar Region. Eleven camera traps were sequentially deployed at 31 locations: 12 in the Ertis Ormany State Forest Nature Reserve and 19 in the Ertis River floodplain near the village of Chernoretsk and the cities of Pavlodar and Aksu, mainly along animal trails and near water sources. Total sampling effort was 998 camera‐trap days, including 554 camera‐trap days in forest ecosystems and 444 camera‐trap days in floodplain ecosystems.

Photographs and videos were processed using Timelapse (Greenberg et al. 2019), and species were identified visually. To avoid repeated records, consecutive observations of the same species at the same location were considered independent when separated by at least 5 min (Suárez‐Tangil and Rodríguez 2021). Camera‐trap monitoring yielded 822 independent mammal records representing 11 species. Camera‐trap data were standardized using Darwin Core terms in accordance with GBIF recommendations for the management and publication of camera‐trap data (Reyserhove et al. 2023) and were published through GBIF as an open dataset in 2026 (Sergazinova 2026).

Potential habitat suitability was modeled for 14 species: *Alces alces* , *Sus scrofa* , *Capreolus pygargus* , *Canis lupus* , *Lynx lynx* , *Vulpes vulpes* , *Vulpes corsac* , *Meles leucurus* , *Mustela eversmanii* , *Mustela erminea* , *Marmota bobak* , *Sciurus vulgaris* , *Ondatra zibethicus* , and *Lepus timidus* .

Population density was calculated from officially obtained departmental materials containing the original quantitative results of species‐specific surveys. For each territorial unit, density was calculated from the recorded abundance and the area of the corresponding territory and expressed as individuals per 1000 ha. Winter track‐survey data were used for most terrestrial species. For species for which this method was not applicable, specialized survey methods were used: *Ondatra zibethicus* was assessed by the number of occupied burrows, whereas *Marmota bobak* was assessed from spring visual counts of individuals and colonies. Because survey methods differed among species, absolute density values were not considered fully comparable across species, and the relationship between predicted habitat suitability and density was analyzed separately for each species.

Density class intervals on the maps were defined separately for each species because absolute density values differed substantially among species and were not directly comparable owing to the use of species‐specific survey methods.

Environmental predictors included bioclimatic variables, topographic characteristics, and vegetation‐cover metrics. Temperature‐ and precipitation‐related bioclimatic variables were obtained from WorldClim v2.1 (WorldClim 2026) and represent climatological means for 1970–2000. Topographic characteristics were derived from the SRTM digital elevation model (CGIAR‐CSI) for 2000 (CGIAR‐CSI SRTM 2026). Vegetation condition was characterized using the Normalized Difference Vegetation Index (NDVI), calculated from Landsat 8 multispectral data obtained through the USGS ESPA service (2026). Images from July 2025 were used to calculate NDVI according to the standard relationship between reflectance in the near‐infrared (NIR) and red (RED) spectral bands.

Before MaxEnt modeling, all environmental predictor layers were clipped to a common modeling extent. Because the source datasets differed in spatial resolution, all rasters were transformed to a common coordinate reference system, spatial extent, aligned raster grid, and working spatial resolution of 2.5 arc‐minutes (0.0416667°). Finer‐resolution layers, including NDVI, were resampled to the working grid of the climatic predictors. Consequently, all MaxEnt input rasters had identical dimensions (142 × 107 cells), spatial extent, and grid alignment. This procedure represented standard spatial‐data harmonization and resampling rather than interpolation of climatic data.

Environmental predictors were selected iteratively and separately for each species. An initial model was first fitted using the full set of ecologically justified variables, after which predictors with minimal relative contribution (percent contribution) and variables with overlapping ecological interpretations were sequentially removed. After each step, the model was rerun and the contributions of the remaining variables were reassessed. The final number of predictors was not predetermined and ranged from 5 to 10 depending on the species. Formal VIF analysis and a fixed pairwise‐correlation threshold were not applied in the original selection procedure; therefore, the contribution of individual variables was interpreted as their relative importance within a particular MaxEnt model rather than as an independent causal effect.

Potential habitat suitability was modeled using the maximum entropy method in MaxEnt 3.4.4. Automatic feature classes and a regularization multiplier of 1.0 were used, and the data were randomly divided into training and test subsets at a 75:25 ratio. Because sample sizes were small for several species, the performance metrics of the corresponding models were interpreted cautiously, particularly for *Marmota bobak* (*n* = 13), *Sciurus vulgaris* (*n* = 15), *Lynx lynx* (*n* = 19), and *Alces alces* (*n* = 21). High AUC values based on small samples were not considered, on their own, sufficient evidence of high model reliability.

Model performance was evaluated using Training AUC, Test AUC, the omission rate at the 10th percentile training presence threshold, and the True Skill Statistic (TSS). AUC characterized overall discriminatory ability, TSS provided an additional measure of the balance between sensitivity and specificity, and the omission rate represented the proportion of known occurrences classified by the model as unsuitable. Absolute AUC values were not used for strict cross‐species ranking of model quality because they can be affected by sample size and spatial structure, characteristics of the background area, and ecological niche breadth.

To quantify the correspondence between predicted habitat suitability and observed population density, analyses were conducted separately for each species across 41 territorial units. For each unit, the continuous suitability surface generated by MaxEnt was aggregated by calculating the mean value within the corresponding territorial polygon. Zero‐density values were retained in the analysis; Ertis Ormany and Bayanaul were each treated as a single spatial unit. The relationship between mean predicted suitability and departmental density estimates (individuals/1000 ha) was assessed using Spearman's rank correlation coefficient (*r*_s_). A non‐parametric test was selected because of the distribution of the data and the large number of zero‐density values; statistical significance was evaluated at *p* < 0.05. Using mean suitability provided a consistent way to compare continuous MaxEnt predictions with polygon‐based density data, although it could smooth within‐polygon spatial heterogeneity and local suitability maxima. In addition, differences in the size and configuration of territorial units could affect the degree of smoothing (the modifiable areal unit problem, MAUP), which was considered when interpreting the results.

To assess the spatial independence of the detected relationships, residual spatial autocorrelation was analyzed using Global Moran's *I*, a spatial weights matrix based on Queen contiguity, and 9999 permutations. Of the 41 territorial units, 40 formed a connected spatial system; Bayanaul was a spatial isolate and was therefore excluded only from the Moran's *I* calculation but retained in the Spearman correlation analysis. For *Canis lupus* , *Marmota bobak* , and *Capreolus pygargus* , which showed statistically significant residual spatial autocorrelation, an additional sensitivity analysis using spatially constrained permutations was performed.

Spatial distribution maps and the final cartographic layouts were produced in a GIS environment. Spatial analysis, processing of environmental rasters, and aggregation of predicted suitability within territorial units were performed in QGIS 4.0.2. Comparison of the two independent data types—MaxEnt‐predicted suitability and departmental density estimates—was used to distinguish potential environmental suitability from realized population patterns.

## 3 Results

Modeling produced predicted habitat‐suitability maps for 14 game mammal species. The models differed in discriminatory ability, the composition of leading environmental predictors, and the degree of correspondence between predicted suitability and observed population density.

### 3.1 Evaluation of Species Distribution Models

The discriminatory ability of the MaxEnt models varied among species. Across the 14 species, Test AUC ranged from 0.538 to 0.992 and TSS from 0.220 to 0.984 (Table 2). The highest Test AUC values were obtained for *Sciurus vulgaris* (0.992 ± 0.003), *Lynx lynx* (0.969 ± 0.015), *Sus scrofa* (0.945 ± 0.017), and *Alces alces* (0.920 ± 0.064). The *Canis lupus* model also showed relatively high discriminatory ability (Test AUC = 0.838 ± 0.044). The lowest Test AUC values were recorded for *Mustela eversmanii* (0.538 ± 0.111) and *Vulpes corsac* (0.553 ± 0.088). The remaining species showed intermediate values.

**TABLE 2:** Assessment of the quality of species distribution models (MaxEnt).

No.	Species	*n* (train/test)	Training AUC	Test AUC ± SD	Test omission rate (10% TP)	TSS	Threshold rule	Number of predictors	Spearman *r*_s_	*p*	Moran *I*	*p* Moran
1	*Alces alces*	16/5	0.972	0.920 ± 0.064	0.200	0.600	Equal test sensitivity & specificity	5	0.3729	0.016	0.0649	0.1989
2	*Sus scrofa*	27/9	0.937	0.945 ± 0.017	0.000	0.778	Equal test sensitivity & specificity	5	0.4943	0.001	0.1628	0.0646
3	*Capreolus pygargus*	142/47	0.845	0.748 ± 0.038	0.234	0.320	Equal test sensitivity & specificity	9	0.5248	< 0.001	0.2281	0.0165
4	*Canis lupus*	30/9	0.863	0.838 ± 0.044	0.111	0.556	Equal test sensitivity & specificity	6	0.7378	< 0.001	0.2291	0.0164
5	*Lynx lynx*	15/4	0.990	0.969 ± 0.015	0.500	0.93	Maximum test sensitivity + specificity	7	0.3733	0.016	0.0623	0.2251
6	*Vulpes vulpes*	58/19	0.750	0.685 ± 0.064	0.263	0.33	Maximum test sensitivity + specificity	6	0.4487	0.003	−0.0804	0.3158
7	*Vulpes corsac*	19/6	0.848	0.553 ± 0.088	0.667	0.25	Maximum test sensitivity + specificity	8	0.3994	0.010	0.0059	0.7637
8	*Meles leucurus*	41/13	0.857	0.725 ± 0.069	0.385	0.384	Equal test sensitivity & specificity	8	0.6100	< 0.001	0.1757	0.0554
9	*Mustela eversmanii*	24/8	0.772	0.538 ± 0.111	0.250	0.22	Maximum test sensitivity + specificity	5	0.2878	0.068	0.1208	0.1664
10	*Mustela erminea*	43/14	0.830	0.700 ± 0.077	0.214	0.286	Equal test sensitivity & specificity	10	0.5417	< 0.001	−0.0037	0.8290
11	*Marmota bobak*	10/3	0.930	0.809 ± 0.136	0.333	0.334	Equal test sensitivity & specificity	8	0.5191	< 0.001	0.3740	0.0002
12	*Sciurus vulgaris*	12/3	0.966	0.992 ± 0.003	0.000	0.984	Equal test sensitivity & specificity	8	0.3640	0.019	0.0501	0.2941
13	*Ondatra zibethicus*	37/12	0.893	0.762 ± 0.057	0.500	0.334	Equal test sensitivity & specificity	8	0.5112	< 0.001	0.0560	0.4393
14	*Lepus timidus*	55/18	0.807	0.688 ± 0.056	0.389	0.222	Equal test sensitivity & specificity	10	0.4089	0.008	0.1674	0.0662

Given the sample sizes, model results for *Marmota bobak* (*n* = 13), *Sciurus vulgaris* (*n* = 15), *Lynx lynx* (*n* = 19), and *Alces alces* (*n* = 21) were interpreted with particular caution. High Test AUC and TSS values for some of these species were not considered sufficient evidence of high model reliability because small sample sizes can increase uncertainty in estimates of predictive performance. Additional interspecific analyses detected no statistically significant relationship between the total number of occurrence records and Test AUC (Spearman's *r*_s_ = −0.508; *p* = 0.064), between sample size and the suitability–density correlation coefficient (*r*_s_ = 0.481; *p* = 0.081), or between Test AUC and the strength of the suitability–density association (*r*_s_ = −0.024; *p* = 0.935). Thus, no statistically significant interspecific relationships were detected among sample size, model discriminatory ability, and the degree of suitability–density correspondence, although the small number of species analyzed limits the statistical power of this comparison.

### 3.2 The Contribution of Environmental Predictors

The relative contribution of environmental variables differed among species; however, temperature‐ and precipitation‐related bioclimatic variables predominated among the leading predictors in most models (Table 3). BIO1 (annual mean temperature), BIO4 (temperature seasonality), BIO5 (maximum temperature of the warmest month), BIO7 (annual temperature range), and BIO10 (mean temperature of the warmest quarter) were particularly frequent among the most important variables.

**TABLE 3:** Contribution of the four most significant predictors in the MaxEnt models, %.

№	Species	Predictor 1	Predictor 2	Predictor 3	Predictor 4
1	*Alces alces*	BIO4 (43.7) (temperature seasonality)	BIO13 (39.1) (precipitation of wettest month)	BIO10 (8.1) (mean temperature of warmest quarter)	BIO5 (6.7) (max temperature of warmest month)
2	*Sus scrofa*	BIO5 (33.1) (max temperature of warmest month)	REL (22.3) (relief index)	BIO10 (21.4) (mean temperature of warmest quarter)	BIO4 (13.2) (temperature seasonality)
3	*Capreolus pygargus*	BIO5 (50.0) (max temperature of warmest month)	NDVI (16.2) (Normalized Difference Vegetation Index)	BIO3 (9.8) (изотермальность/isothermality)	BIO12 (7.3) (annual precipitation)
4	*Canis lupus*	BIO11 (47.0) (mean temperature of coldest quarter)	BIO8 (22.0) (mean temperature of wettest quarter)	BIO13 (18.3) (precipitation of wettest month)	BIO2 (8.0) (mean diurnal range)
5	*Lynx lynx*	BIO7 (44.6) (annual temperature range)	BIO4 (23.4) (temperature seasonality)	BIO13 (11.5) (precipitation of wettest month)	BIO3 (7.2) (isothermality)
6	*Vulpes vulpes*	BIO1 (39.3) (annual mean temperature)	BIO7 (22.9) (annual temperature range)	REL (16.8) (relief index)	NDVI (14.6) (Normalized Difference Vegetation Index)
7	*Vulpes corsac*	BIO1 (36.2) (annual mean temperature)	BIO3 (17.2) (isothermality)	BIO7 (12.6) (annual temperature range)	BIO13 (10.2) (precipitation of wettest month)
8	*Meles leucurus*	BIO7 (36.7) (annual temperature range)	BIO10 (28.1) (mean temperature of warmest quarter)	BIO14 (7.4) (precipitation of driest month)	BIO13 (7.0) (precipitation of wettest month)
9	*Mustela eversmanii*	BIO1 (45.9) (annual mean temperature)	BIO4 (40.4) (temperature seasonality)	BIO12 (13.6) (annual precipitation)	BIO15 (0.1) (precipitation seasonality)
10	*Mustela erminea*	BIO5 (39.0) (max temperature of warmest month)	NDVI (20.6) (Normalized Difference Vegetation Index)	BIO10 (11.3) (mean temperature of warmest quarter)	BIO15 (9.1) (precipitation seasonality)
11	*Marmota bobak*	NDVI (35.2) (Normalized Difference Vegetation Index)	BIO14 (34.7) (precipitation of driest month)	REL (18) (relief index)	BIO7 (6.1) (annual temperature range)
12	*Sciurus vulgaris*	BIO4 (72.4) (temperature seasonality)	BIO7 (14.4) (annual temperature range)	BIO13 (10.2) (precipitation of wettest month)	BIO9 (1.3) (mean temperature of driest quarter)
13	*Ondatra zibethicus*	BIO5 (52.4) (max temperature of warmest month)	NDVI (28.0) (Normalized Difference Vegetation Index)	BIO11 (6.1) (mean temperature of coldest quarter)	BIO4 (5.7) (temperature seasonality)
14	*Lepus timidus*	BIO10 (60.7) (mean temperature of warmest quarter)	BIO16 (10.5) (precipitation of wettest quarter)	NDVI (8.6) (Normalized Difference Vegetation Index)	REL (7.8) (relief index)

A pronounced dominance of one or two predictors was observed for several species. For *Sciurus vulgaris* , the relative contribution of BIO4 was 72.4%; for *Lepus timidus* , BIO10 contributed 60.7%; for *Ondatra zibethicus* , BIO5 contributed 52.4%; for *Capreolus pygargus* , BIO5 contributed 50.0%; and for *Canis lupus* , BIO11 contributed 47.0%. For *Alces alces* , the combined relative contribution of the two leading predictors–BIO4 (43.7%) and BIO13 (39.1%)–was 82.8%.

In some models, NDVI and the relief index were also among the leading predictors. The relative contribution of NDVI was 35.2% for *Marmota bobak* , 28.0% for *Ondatra zibethicus* , 20.6% for *Mustela erminea* , and 16.2% for *Capreolus pygargus* . The relief index was among the leading predictors for *Sus scrofa* (22.3%), *Marmota bobak* (18.0%), and *Vulpes vulpes* (16.8%).

Overall, bioclimatic variables made the largest relative contribution in most models, whereas NDVI and the relief index were among the leading predictors only for some species. Because residual collinearity among environmental predictors may have remained, percent contribution values were interpreted as measures of relative variable importance within the corresponding MaxEnt model rather than as estimates of independent causal effects.

### 3.3 Relationship Between Predicted Habitat Suitability and Population Density

Predicted suitability and departmental density estimates were compared on a common spatial basis comprising 41 territorial units. Spearman's correlation coefficients ranged from 0.288 to 0.738. In the initial analysis, a statistically significant positive relationship was detected for 13 of the 14 species; the exception was *Mustela eversmanii* (*r*_s_ = 0.288; *p* = 0.068). For species with statistically significant residual spatial autocorrelation, the robustness of these results was additionally assessed using spatially constrained permutations.

Species‐specific spatial comparisons are presented sequentially for *Alces alces* (Figure 2), *Sus scrofa* (Figure 3), *Capreolus pygargus* (Figure 4), *Canis lupus* (Figure 5), *Lynx lynx* (Figure 6), *Vulpes vulpes* (Figure 7), *Vulpes corsac* (Figure 8), *Meles leucurus* (Figure 9), *Mustela eversmanii* (Figure 10), *Mustela erminea* (Figure 11), *Marmota bobak* (Figure 12), *Sciurus vulgaris* (Figure 13), *Ondatra zibethicus* (Figure 14), and *Lepus timidus* (Figure 15).

**FIGURE 2:**
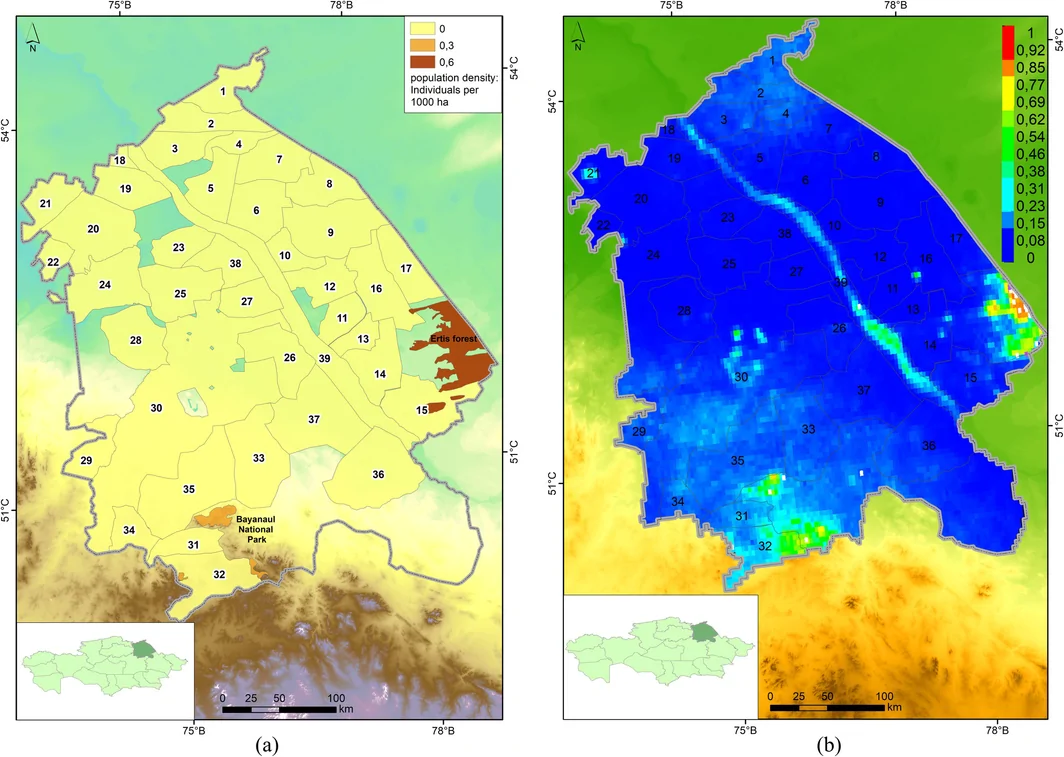
Comparative spatial analysis of population density and predicted habitat suitability for *Alces alces* : (a) spatial distribution of population density based on traditional census data (individuals/1000 ha); (b) predicted habitat suitability based on MaxEnt modeling (suitability index 0–1).

**FIGURE 3:**
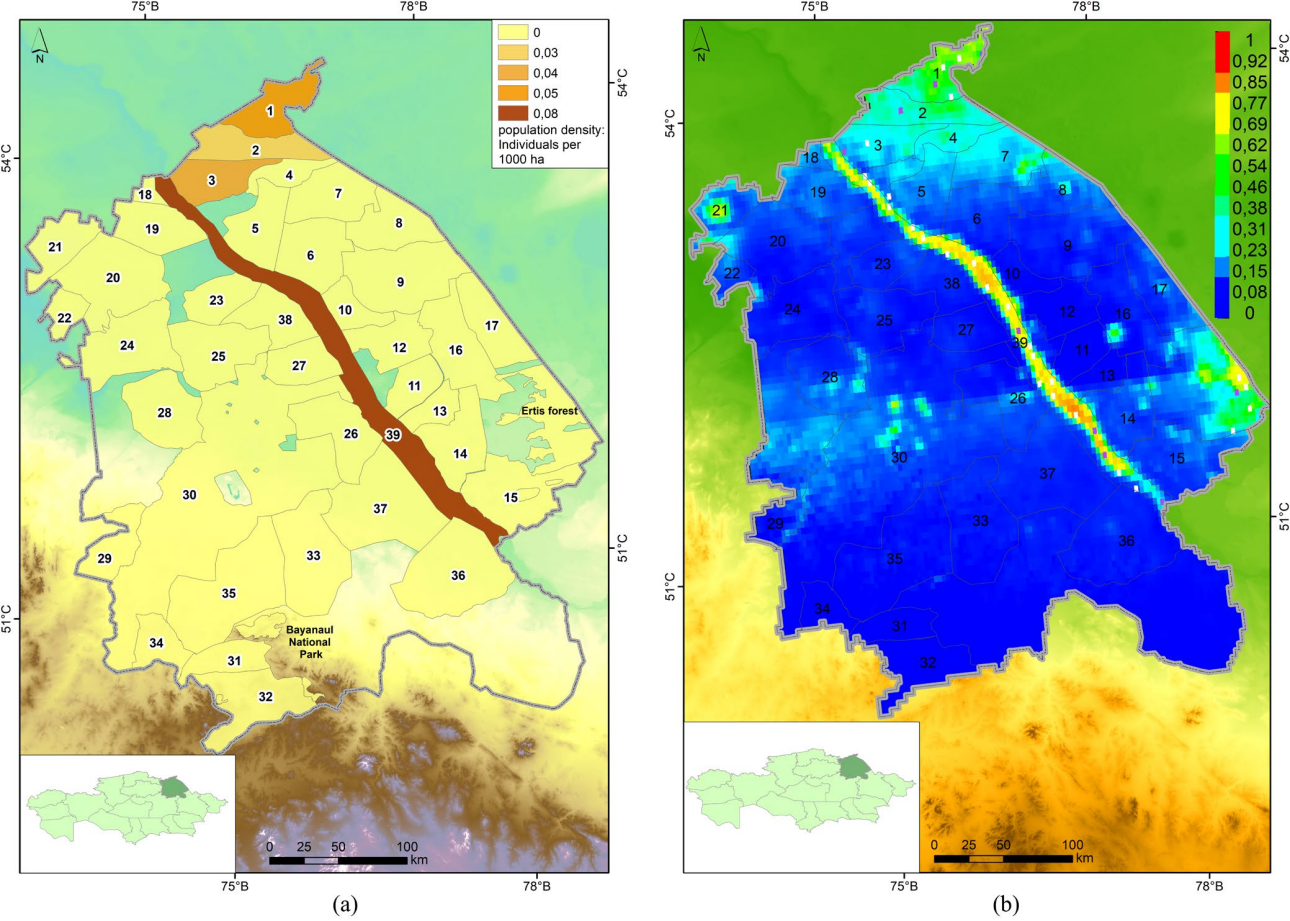
Comparative spatial analysis of population density and predicted habitat suitability for *Sus scrofa* : (a) spatial distribution of population density based on traditional census data (individuals/1000 ha); (b) predicted habitat suitability based on MaxEnt modeling (suitability index 0–1).

**FIGURE 4:**
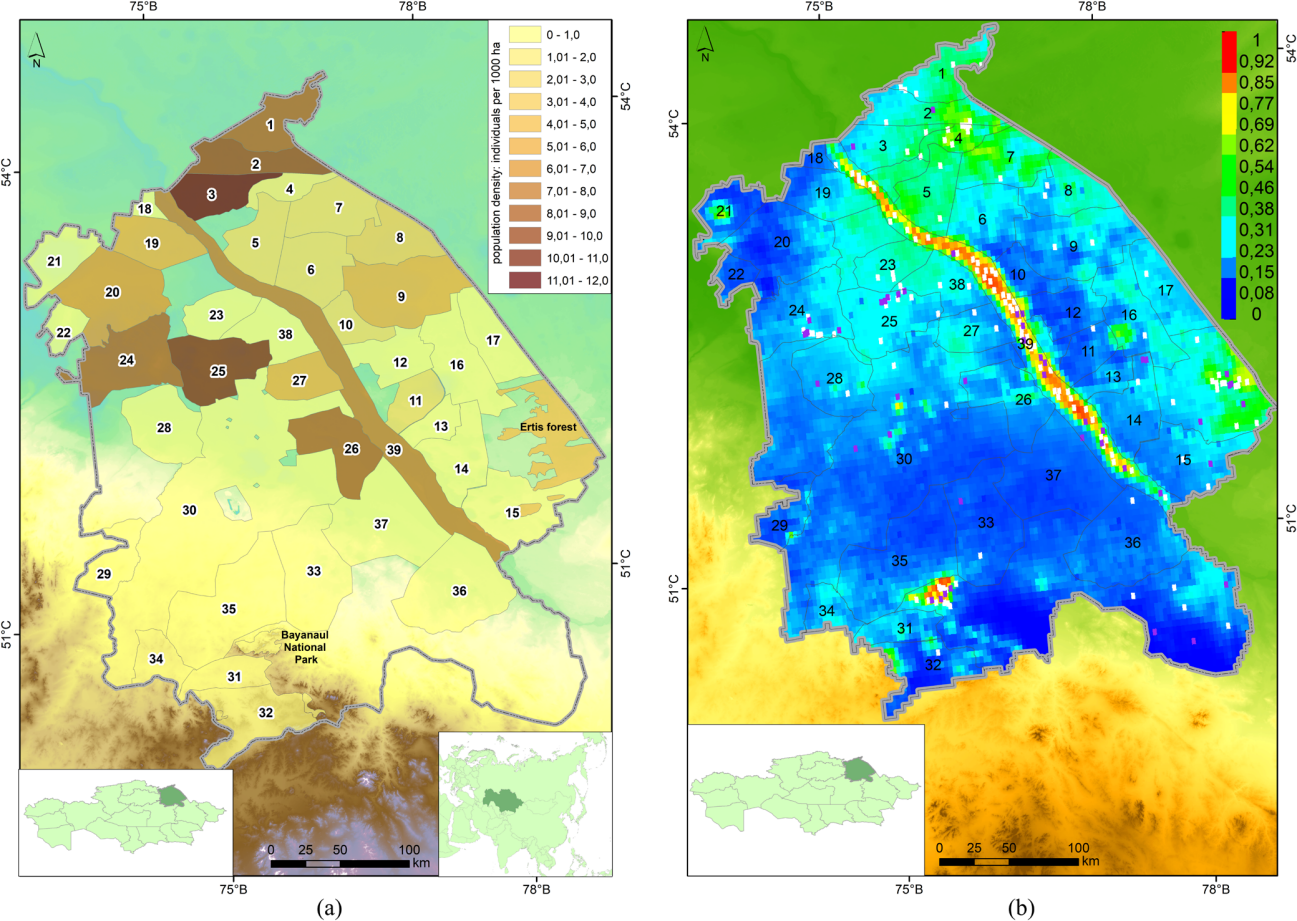
Comparative spatial analysis of population density and predicted habitat suitability for *Capreolus pygargus* : (a) spatial distribution of population density based on traditional census data (individuals/1000 ha); (b) predicted habitat suitability based on MaxEnt modeling (suitability index 0–1).

**FIGURE 5:**
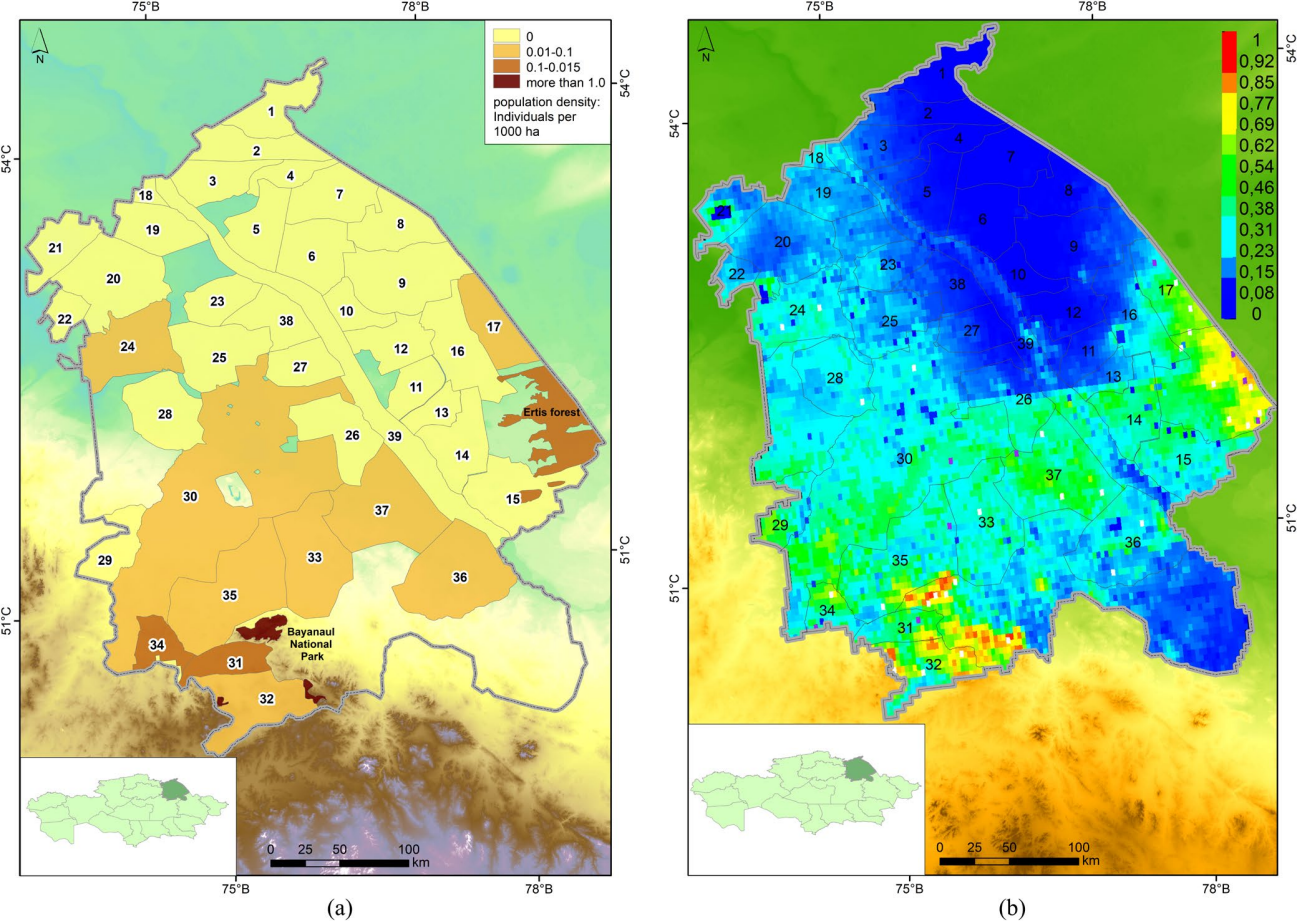
Comparative spatial analysis of population density and predicted habitat suitability for *Canis lupus* : (a) spatial distribution of population density based on traditional census data (individuals/1000 ha); (b) predicted habitat suitability based on MaxEnt modeling (suitability index 0–1).

**FIGURE 6:**
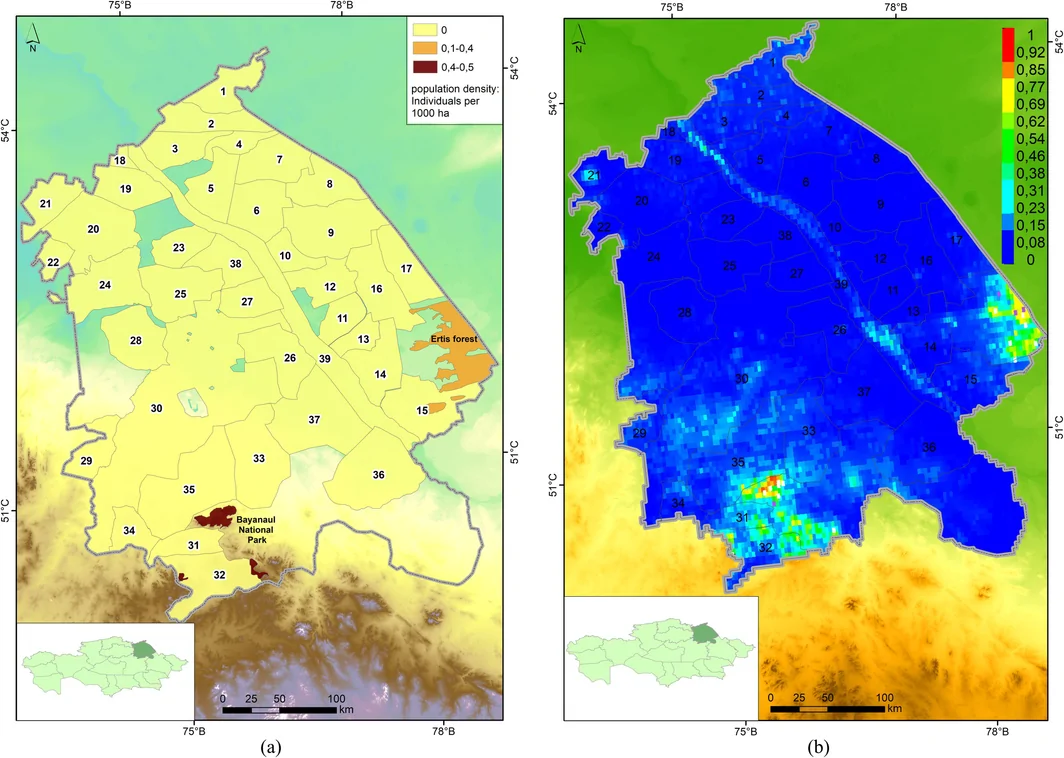
Comparative spatial analysis of population density and predicted habitat suitability for *Lynx lynx* : (a) spatial distribution of population density based on traditional census data (individuals/1000 ha); (b) predicted habitat suitability based on MaxEnt modeling (suitability index 0–1).

**FIGURE 7:**
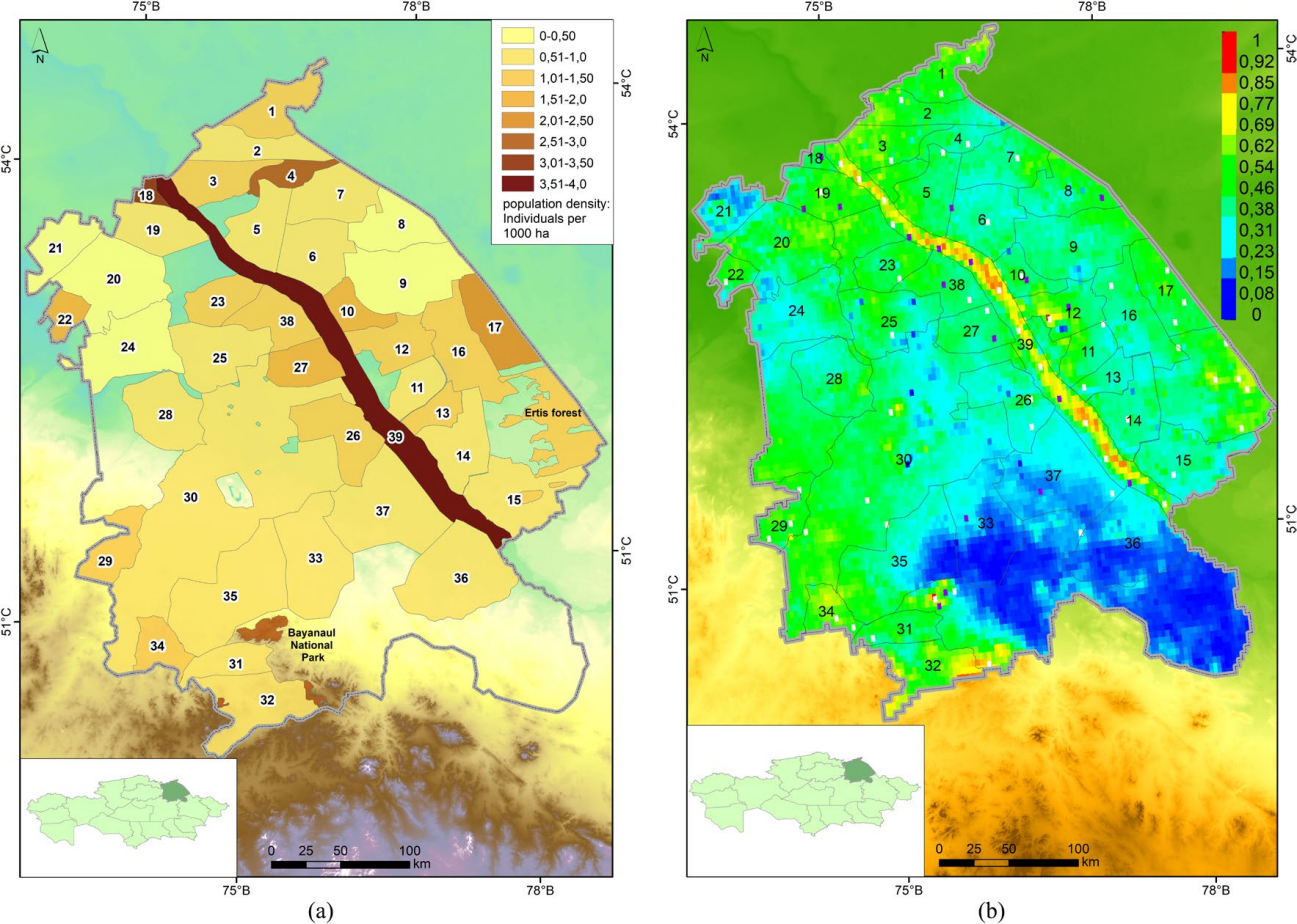
Comparative spatial analysis of population density and predicted habitat suitability for *Vulpes vulpes* : (a) spatial distribution of population density based on traditional census data (individuals/1000 ha); (b) predicted habitat suitability based on MaxEnt modeling (suitability index 0–1).

**FIGURE 8:**
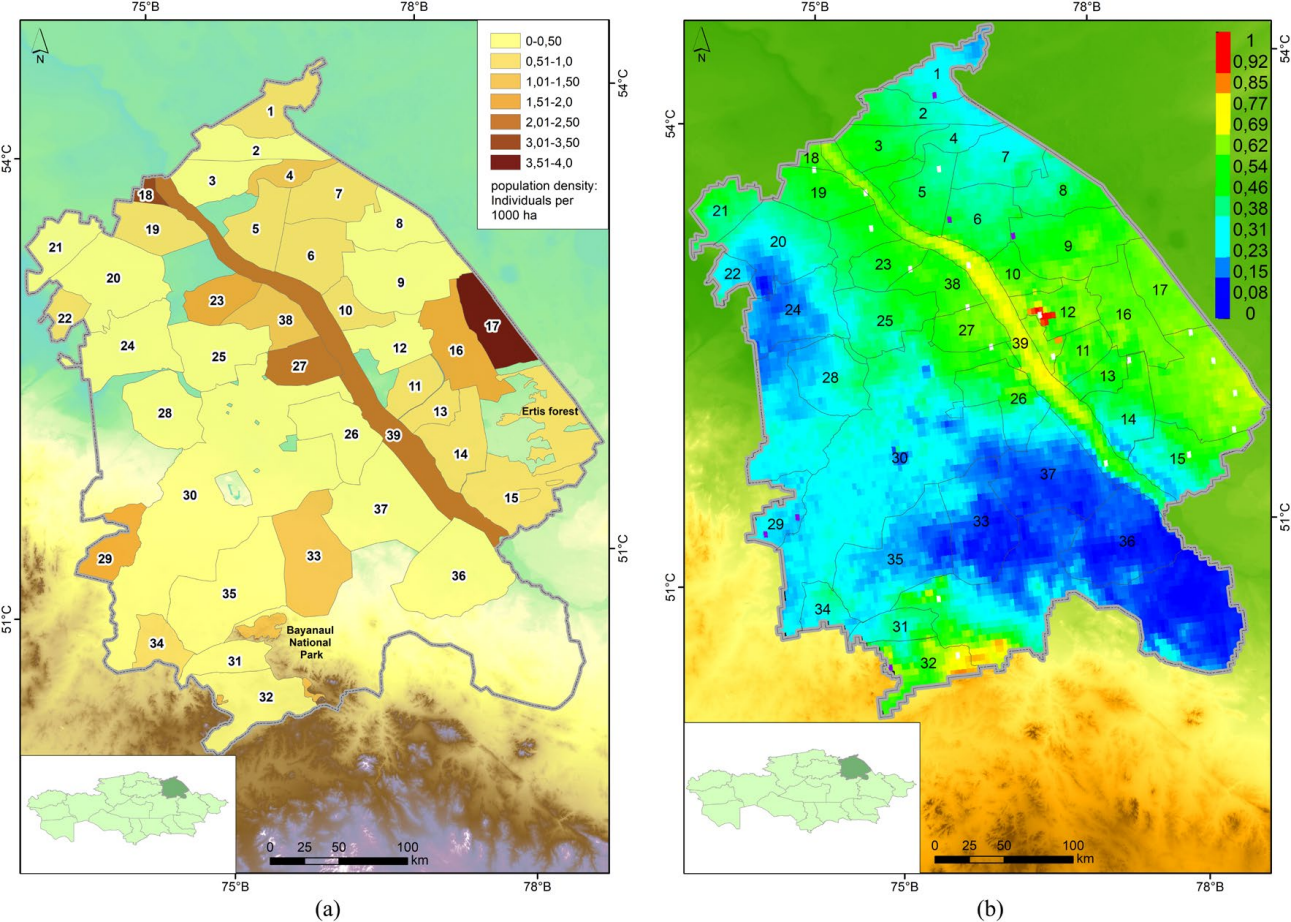
Comparative spatial analysis of population density and predicted habitat suitability for *Vulpes corsac* : (a) spatial distribution of population density based on traditional census data (individuals/1000 ha); (b) predicted habitat suitability based on MaxEnt modeling (suitability index 0–1).

**FIGURE 9:**
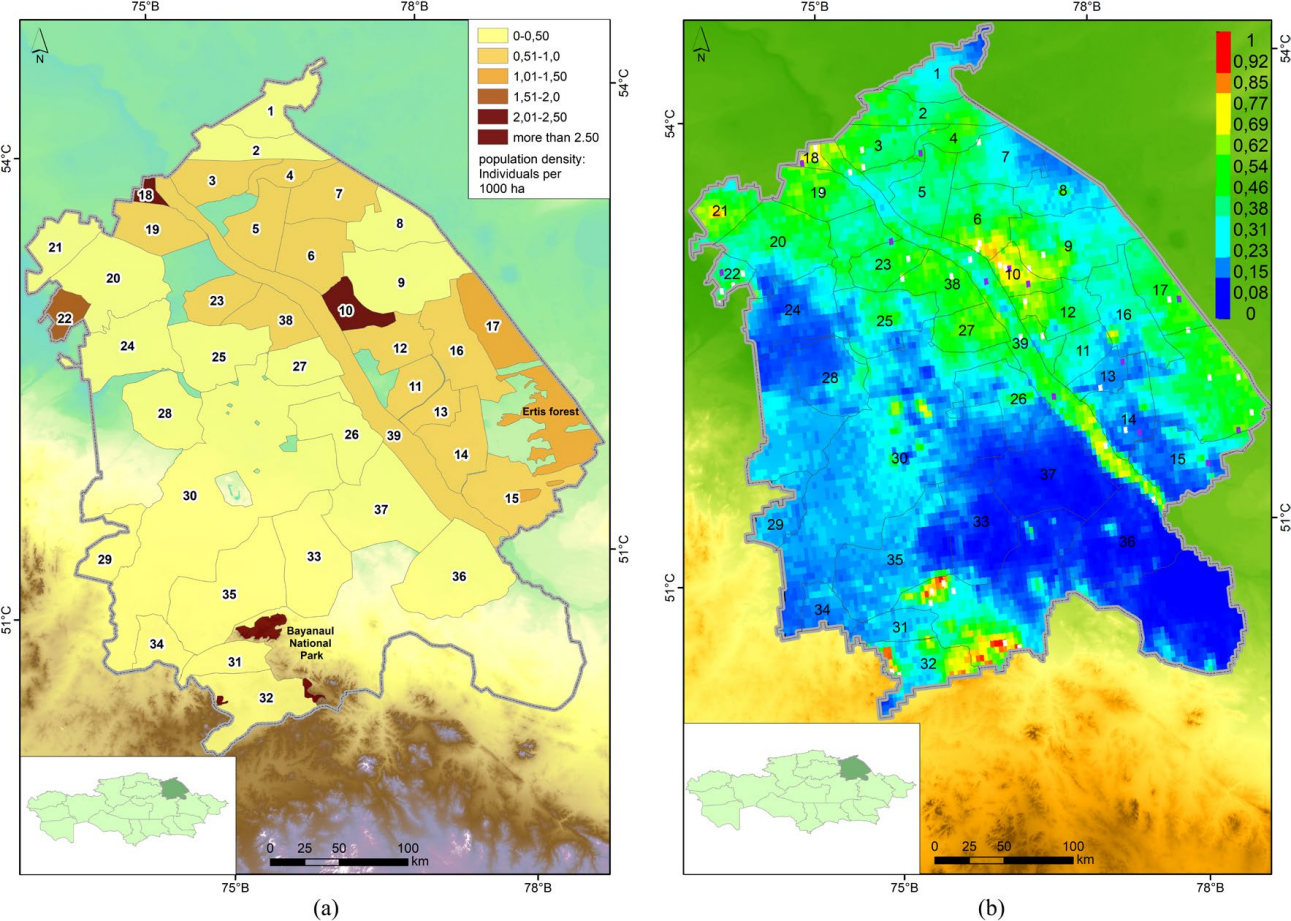
Comparative spatial analysis of population density and predicted habitat suitability for *Meles leucurus* : (a) spatial distribution of population density based on traditional census data (individuals/1000 ha); (b) predicted habitat suitability based on MaxEnt modeling (suitability index 0–1).

**FIGURE 10:**
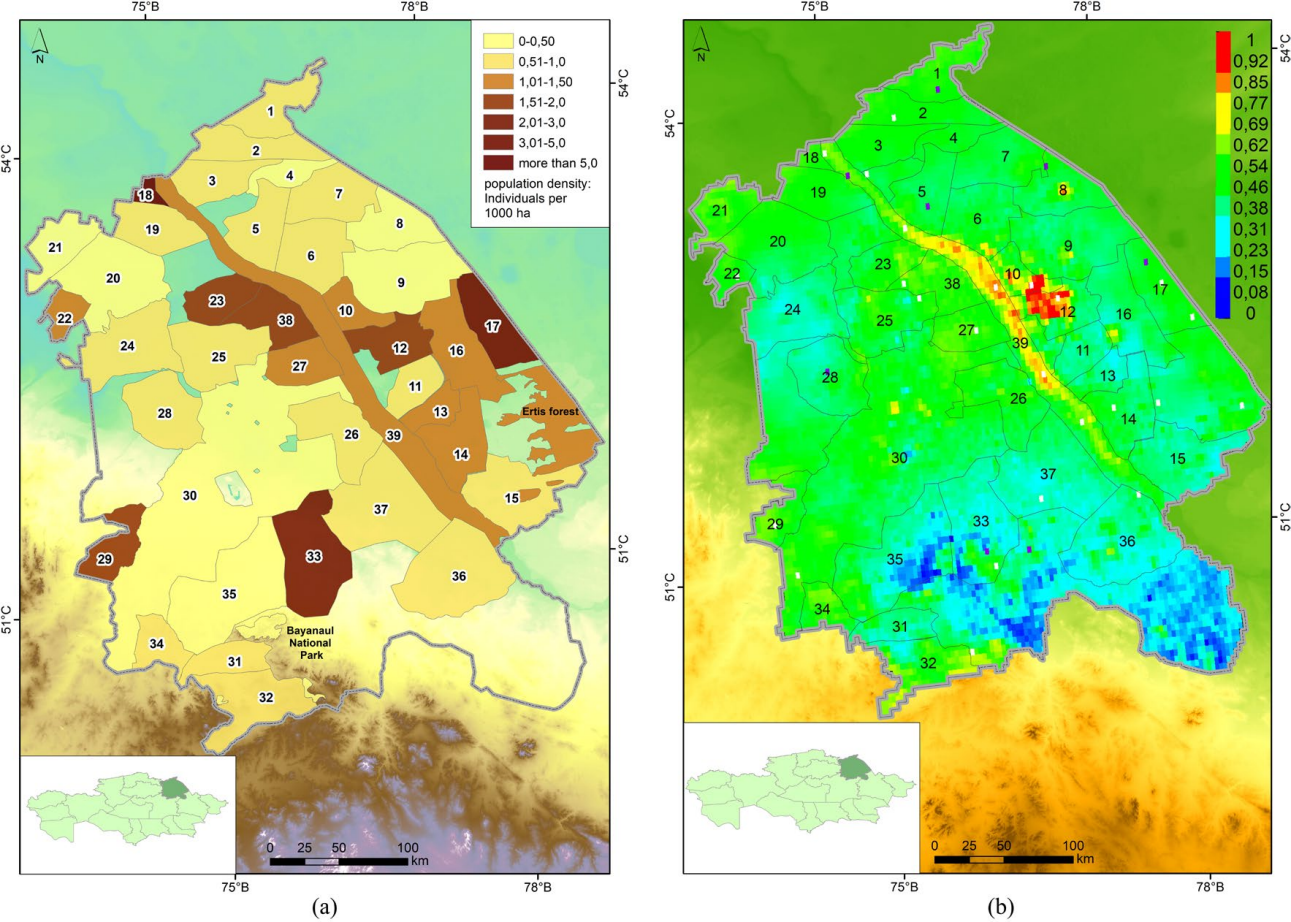
Comparative spatial analysis of population density and predicted habitat suitability for *Mustela eversmanii* : (a) spatial distribution of population density based on traditional census data (individuals/1000 ha); (b) predicted habitat suitability based on MaxEnt modeling (suitability index 0–1).

**FIGURE 11:**
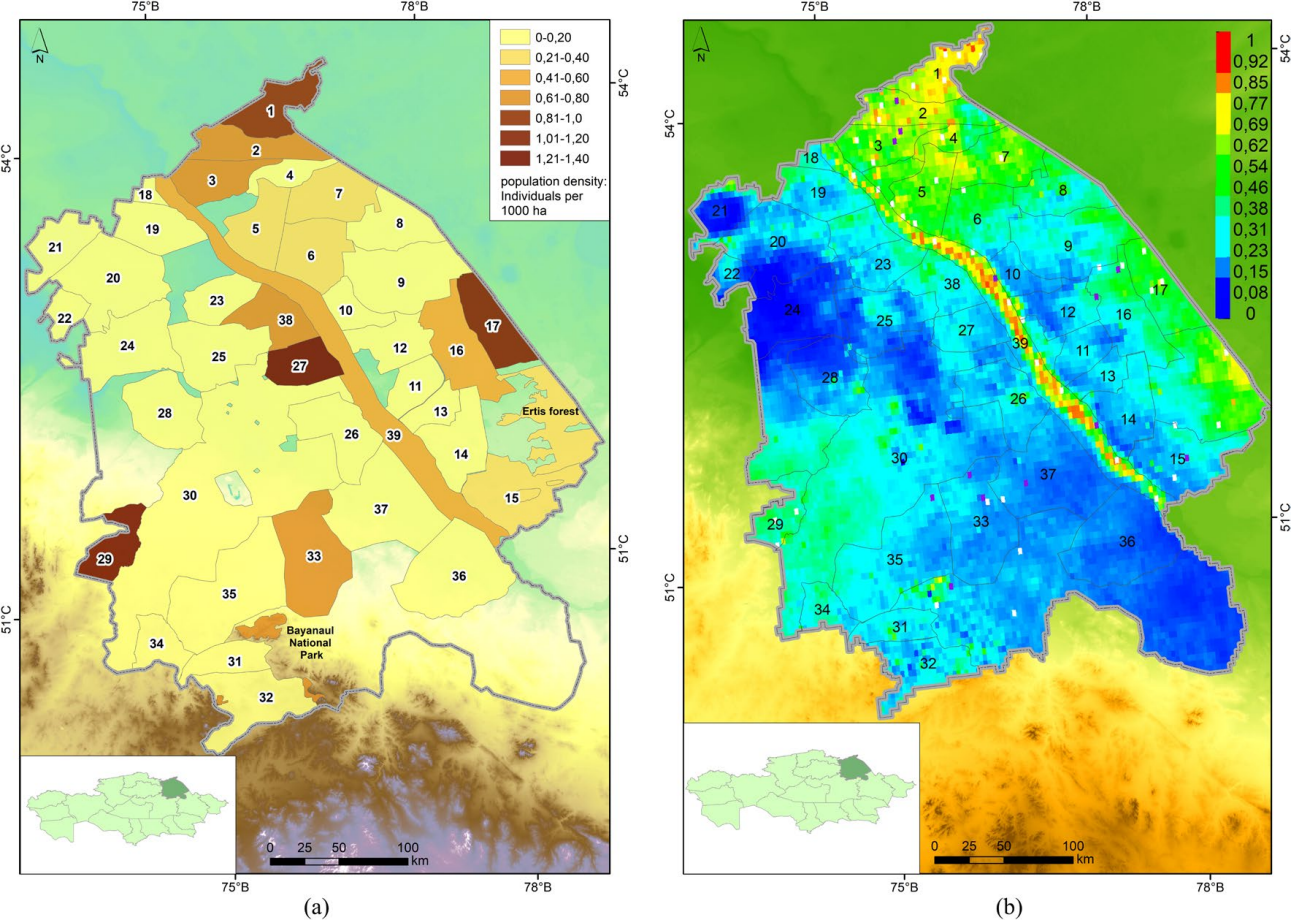
Comparative spatial analysis of population density and predicted habitat suitability for *Mustela erminea* : (a) spatial distribution of population density based on traditional census data (individuals/1000 ha); (b) predicted habitat suitability based on MaxEnt modeling (suitability index 0–1).

**FIGURE 12:**
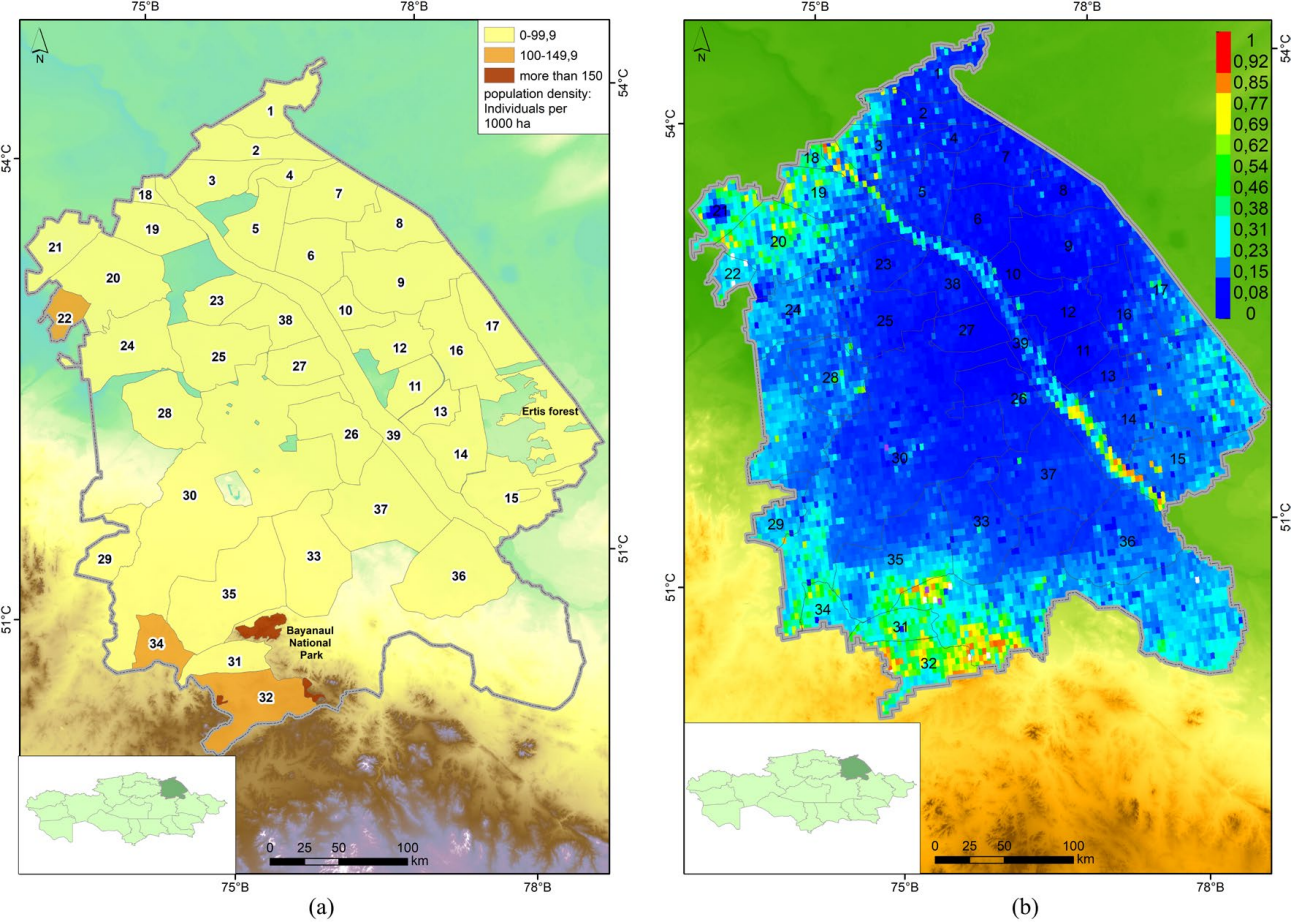
Comparative spatial analysis of population density and predicted habitat suitability for *Marmota bobak* : (a) spatial distribution of population density based on traditional census data (individuals/1000 ha); (b) predicted habitat suitability based on MaxEnt modeling (suitability index 0–1).

**FIGURE 13:**
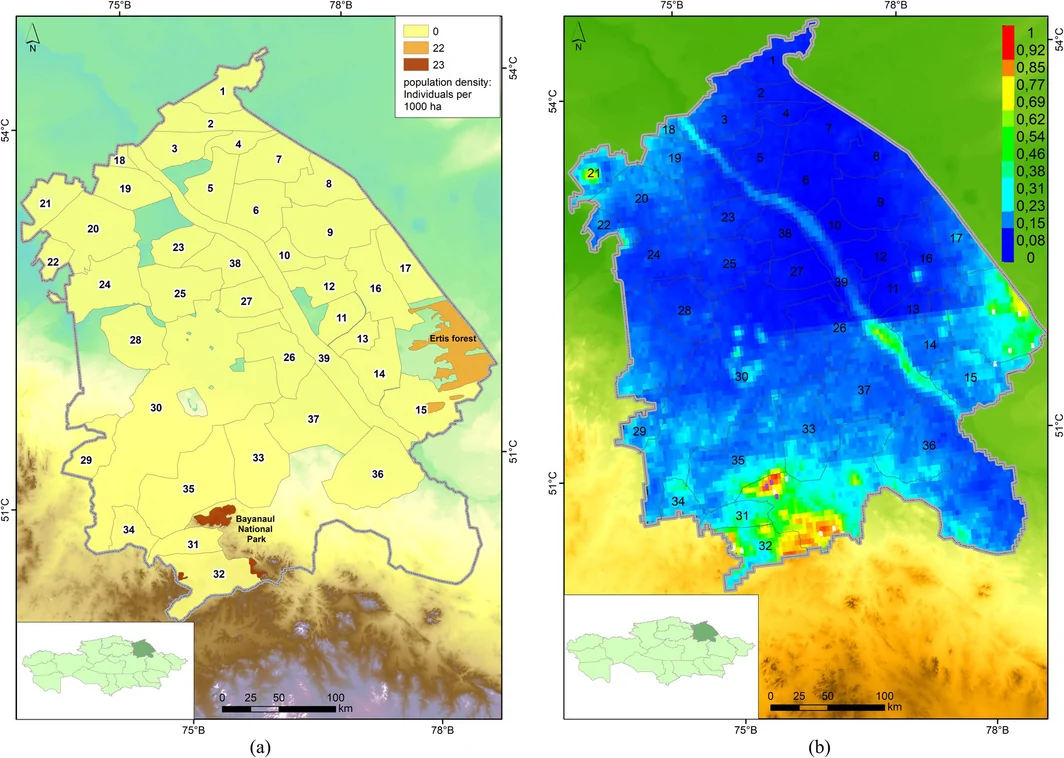
Comparative spatial analysis of population density and predicted habitat suitability for *Sciurus vulgaris* : (a) spatial distribution of population density based on traditional census data (individuals/1000 ha); (b) predicted habitat suitability based on MaxEnt modeling (suitability index 0–1).

**FIGURE 14:**
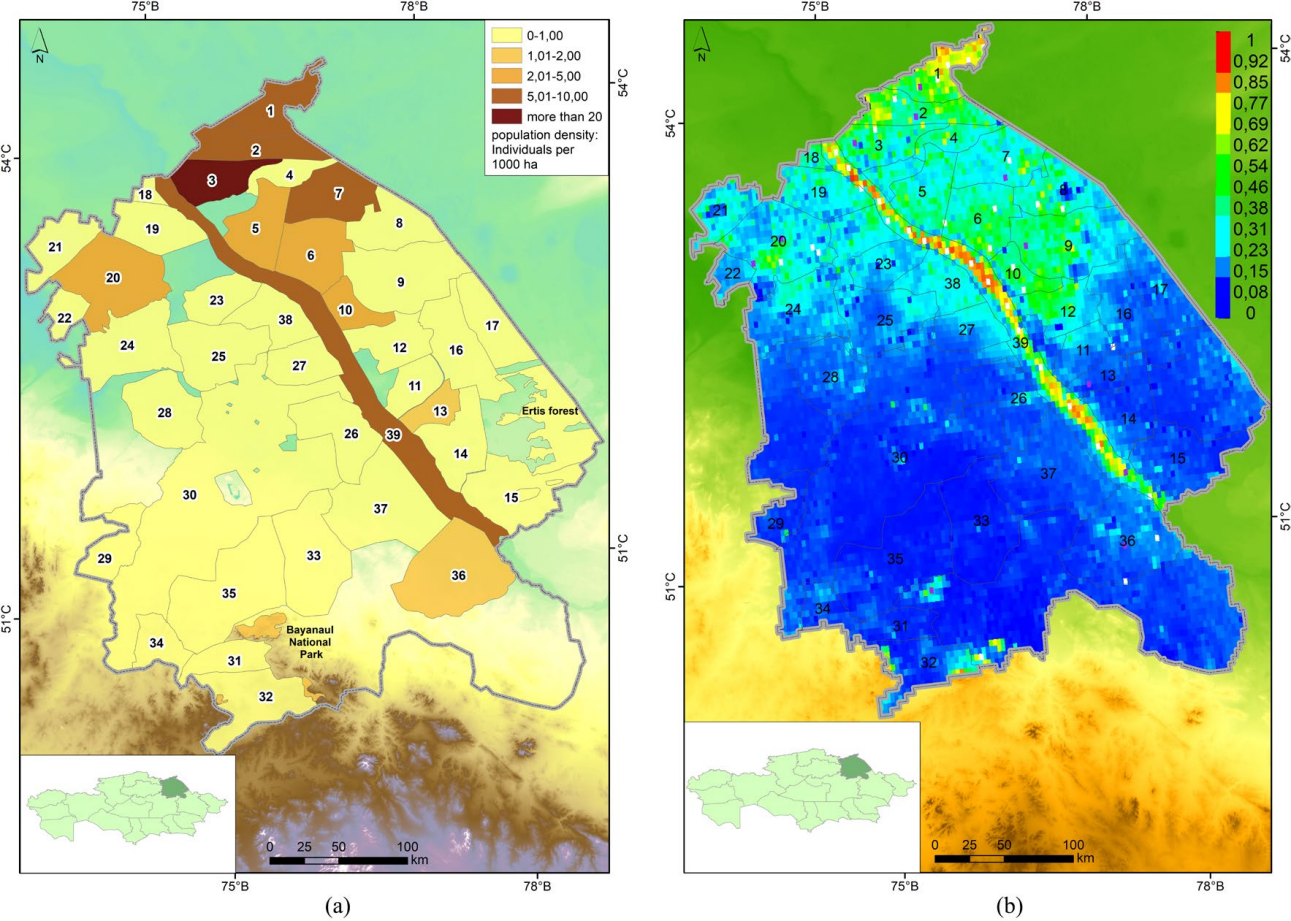
Comparative spatial analysis of population density and predicted habitat suitability for *Ondatra zibethicus* : (a) spatial distribution of population density based on traditional census data (individuals/1000 ha); (b) predicted habitat suitability based on MaxEnt modeling (suitability index 0–1).

**FIGURE 15:**
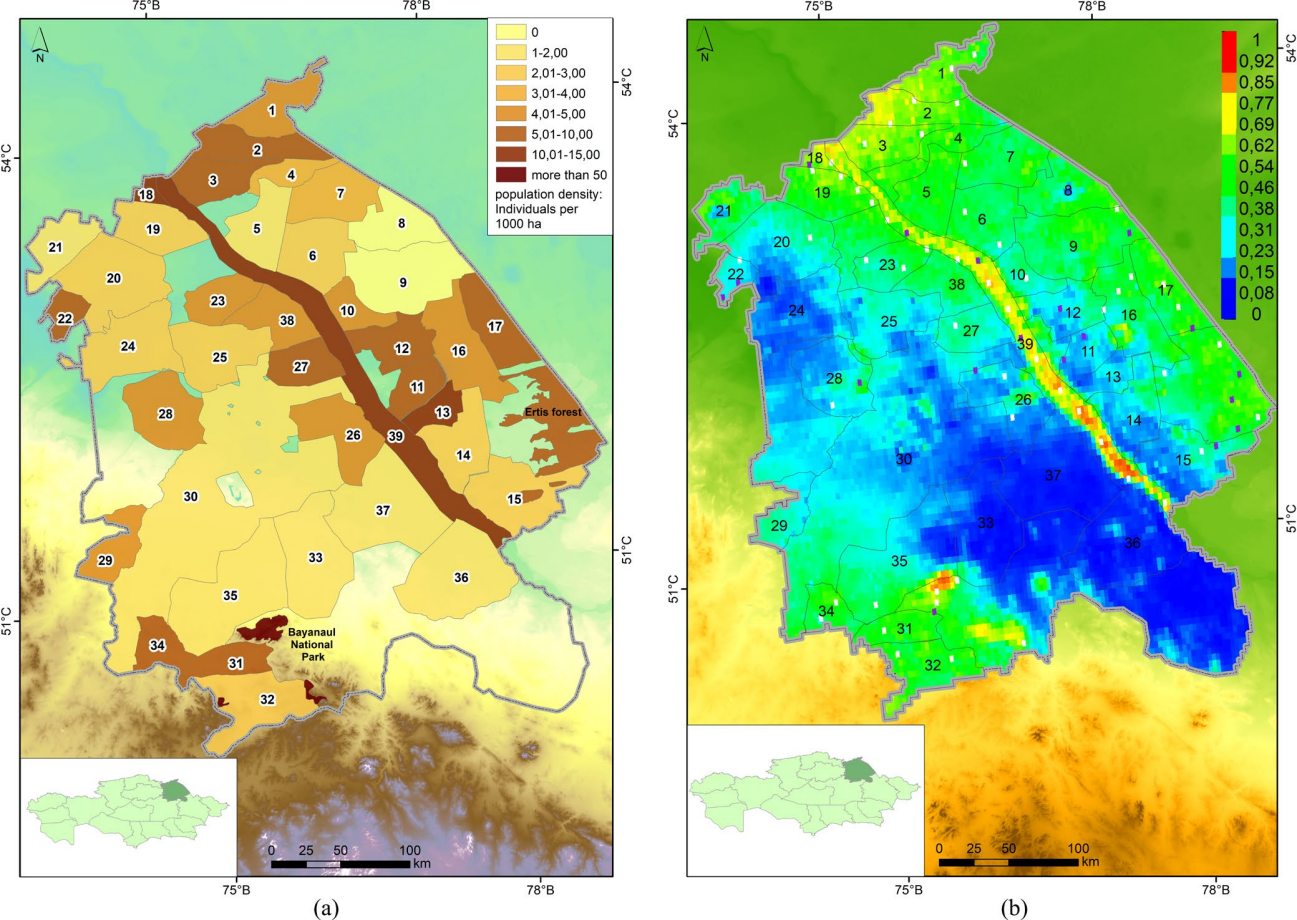
Comparative spatial analysis of population density and predicted habitat suitability for *Lepus timidus* : (a) spatial distribution of population density based on traditional census data (individuals/1000 ha); (b) predicted habitat suitability based on MaxEnt modeling (suitability index 0–1).

The strongest positive relationships between predicted suitability and observed density were found for *Canis lupus* (*r*_s_ = 0.738) and *Meles leucurus* (*r*_s_ = 0.610). For the remaining species with statistically significant initial correlations, *r*_s_ values ranged from 0.364 to 0.542. Full correlation coefficients and significance levels are provided in Table 2.

Spatial comparison of the maps showed that, in some cases, MaxEnt identified broader areas of potential suitability than the areas of elevated observed density. The most pronounced mismatch was observed for *Mustela eversmanii* and *Vulpes corsac* . For *Marmota bobak* , predicted suitable areas were also broader than the spatially restricted cores of observed density, whereas for *Ondatra zibethicus* , the observed density distribution was more fragmented than the predicted suitability pattern.

No direct correspondence was observed between the discriminatory ability of the MaxEnt models and the strength of the suitability–density relationship. *Sciurus vulgaris* and *Lynx lynx* had some of the highest Test AUC and TSS values, yet the relationship between predicted suitability and observed density was relatively weak for these species (*r*_s_ = 0.364 and 0.373, respectively). In contrast, *Canis lupus* and *Meles leucurus* showed stronger suitability–density relationships (*r*_s_ = 0.738 and 0.610, respectively) despite less extreme model‐performance metrics.

Analysis of residual spatial structure revealed no statistically significant spatial autocorrelation for 11 of the 14 species. Significant Moran's *I* values were detected for *Canis lupus* (*I* = 0.2291; *p* = 0.0164), *Marmota bobak* (*I* = 0.3740; *p* = 0.0002), and *Capreolus pygargus* (*I* = 0.2281; *p* = 0.0165). For these three species, an additional analysis using spatially constrained permutations was performed. After this analysis, the positive relationship between predicted suitability and density remained statistically significant for *Canis lupus* (*r*_s_ = 0.714; *p* = 0.0007) and *Capreolus pygargus* (*r*_s_ = 0.488; *p* = 0.0198). For *Marmota bobak* , the relationship remained positive but did not reach statistical significance after accounting for spatial structure (*r*_s_ = 0.467; *p* = 0.0615).

Thus, the relationship between MaxEnt‐predicted suitability and observed density differed among species. In the initial correlation analysis, a statistically significant positive relationship was detected for most species; however, after accounting for spatial structure, it was no longer statistically significant for *Marmota bobak* . In addition, the high discriminatory ability of a MaxEnt model did not, by itself, correspond to a stronger suitability–density relationship.

## 4 Discussion

Pavlodar Region provides an informative setting for testing the relationship between predicted habitat suitability and observed population density because ribbon pine forests, the Irtysh floodplain, open steppe, semi‐desert areas, and the mountain‐forest complexes of Bayanaul occur within a single region. This landscape and climatic contrast make it possible to compare species with different ecologies within a common spatial framework. Unlike studies limited to mapping potential ranges, the present study directly compares MaxEnt suitability with independent departmental density estimates, thereby contrasting potential environmental suitability with realized population patterns.

Overall, 1 was supported: a positive suitability‐density relationship was detected for most species, although its strength varied markedly among species. After accounting for spatial structure, the positive relationship remained statistically significant for wolf and roe deer, whereas for bobak marmot it remained positive but did not reach statistical significance. Thus, MaxEnt suitability reflects the environmental potential of an area but cannot be treated as a direct quantitative equivalent of population density. This is consistent with the general view that SDMs primarily characterize potentially suitable conditions, whereas realized distribution and abundance are additionally constrained by biotic interactions, resources, anthropogenic pressure, and population spatial dynamics (Peterson et al. 2011).

Species‐specific differences indicate that a similar macroecological signal can be realized in different ways. For moose, the association with forested and humid habitats is consistent with the known dependence of the species on thermoregulatory conditions, water availability, and temperature constraints (van Beest et al. 2012; Dussault et al. 2004; Murray et al. 2006; Lenarz et al. 2009; Teitelbaum et al. 2021). Wild boar, in contrast, can use a broad range of mosaic, floodplain, agricultural, and disturbed habitats; its distribution is shaped by a combination of climate, water resources, structural complexity, and anthropogenic landscape transformation (Lee et al. 2022; Marin et al. 2024; Lim et al. 2025; Eshtiaghi et al. 2024). Roe deer is associated with transitional and ecotonal habitats, where vegetation structure, landscape heterogeneity, and anthropogenic effects operate alongside thermal conditions (Eom et al. 2023; Zorzi et al. 2022; Ruco and Marucco 2025). These examples show that even where a climatic signal is present, realized density is shaped at a finer spatial scale.

For mammalian predators, the role of factors not directly represented by macroclimatic layers is particularly evident. Wolf spatial structure depends not only on climate and landscape but also on prey distribution, road density, anthropogenic transformation, high mobility, and pack dynamics (van den Bosch et al. 2023; Kudrenko et al. 2023; Reshamwala et al. 2022; Ferreiro‐Arias et al. 2024). For lynx, forest cover, continuity of shelter, and prey availability are important (Filla et al. 2017; Ripari et al. 2022). The broad ecological tolerance of the red fox allows it to use forest, steppe, agricultural, and floodplain habitats, so local density is additionally influenced by food resources, disturbance, and habitat transformation (Allen et al. 2025). For corsac fox, despite its association with open steppe and semi‐desert landscapes, small‐rodent abundance, grazing pressure, local land use, and population mobility may be important (Ahmadpour et al. 2021).

A similar dependence on local resources is evident among mustelids. For badger, macroclimatic conditions provide the broad environmental background, whereas realized occurrence is linked to microtopography, soil type, and the suitability of sites for burrow construction (Bae et al. 2021; Li et al. 2024). For steppe polecat, which showed the weakest and non‐significant suitability‐density relationship, the availability of small mammals, burrow‐habitat structure, and transformation of steppe ecosystems are particularly important; climatic effects may operate indirectly through trophic interactions (Korpela et al. 2014; Kiseleva 2024). Stoat distribution likewise depends strongly on local prey abundance and shelter structure, which are poorly represented by broad‐scale climatic predictors (Granata et al. 2026). Thus, for small mobile predators, the mismatch between potential suitability and realized density is ecologically plausible.

For species strongly dependent on specific habitats, macroecological models likewise do not fully reproduce local density. In bobak marmot, vegetation productivity, moisture, landscape openness, and relief interact with local constraints related to soils, burrow suitability, grazing pressure, and colony history; potentially suitable areas may therefore be broader than actual colony cores (Koshkina et al. 2020). In red squirrel, the clear association with woody vegetation facilitates identification of suitable forest patches, but local abundance depends on stand composition and structure, food resources, and forest disturbance (Zhang et al. 2025). For muskrat, broad suitability is associated with aquatic and riparian habitats, whereas realized density additionally depends on waterbody configuration, the balance between open water and emergent vegetation, and hydrological regime (Melvin and Bowman 2026; Allen and Hoffman 1984). For mountain hare near the southern part of its range, snow regime, duration of snow cover, shelter, and microclimatic conditions are important and contribute to a fragmented realized distribution (Martino et al. 2024; Göransson et al. 2025).

The results do not support a simple «ecological specialist = stronger suitability‐density agreement, generalist = weaker agreement» scheme. Notably, high Test AUC values for lynx and red squirrel were obtained from small occurrence samples but were not accompanied by the strongest density relationships, whereas wolf and badger showed stronger suitability‐density agreement at less extreme discrimination values. Additional interspecific analyses detected no statistically significant relationship between sample size and Test AUC, between sample size and suitability‐density strength, or between Test AUC and the strength of the suitability‐density association. Accordingly, 2 was not supported, and 3 was also not consistently supported: mismatch was pronounced for steppe polecat and evident for corsac fox, but this pattern did not extend unambiguously to all ecologically plastic or steppe species.

The predominance of temperature‐ and precipitation‐related variables among the leading predictors is consistent with the sharply continental climate of the region, where seasonality and temperature extremes define broad limits of environmental suitability. At the same time, the contribution of NDVI and relief for particular species indicates that vegetation productivity and landscape structure complement climatic control. Bioclimatic variables should therefore be interpreted as factors defining the regional environmental background rather than as independent causal mechanisms of local density. This interpretation is consistent with studies emphasizing climatic constraints on mammal distributions (Phillips et al. 2006; Elith et al. 2006; Li et al. 2020; Biancolini et al. 2024; Lawlor et al. 2024).

Several methodological limitations are important for interpreting interspecific differences. First, high performance metrics for several small‐sample species, including lynx, red squirrel, moose, and bobak marmot, should be treated cautiously because limited occurrence data increase uncertainty in estimates of predictive performance. Second, averaging MaxEnt suitability within 41 territorial units smooths local maxima, and the degree of smoothing depends on polygon size. This manifestation of the modifiable areal unit problem (MAUP) is particularly important for species with localized density hotspots and many zero‐density values. For species with predominantly zero or near‐zero density values across territorial units, particularly *Lynx lynx* , the suitability–density correlation contains limited information and should therefore be interpreted cautiously. The resulting correlations therefore describe correspondence at the management‐unit scale rather than a local presence–abundance relationship.

Third, differences among density‐survey methods limit direct cross‐species comparison. Winter track surveys are applicable to most terrestrial species, whereas muskrat was assessed from burrows and bobak marmot from spring visual counts. Interspecific differences may therefore partly reflect detectability and census methodology rather than ecology alone. An additional limitation is the temporal mismatch between the 1970–2000 WorldClim climatology and biological data from 2020 to 2025: the climatic layers represent long‐term background conditions rather than weather during particular survey years. Furthermore, presence‐only data and the omission of some local factors—such as land‐use structure, anthropogenic pressure, and spatial distribution of food resources—limit the ability of MaxEnt to reproduce realized density (Mancini et al. 2025).

From an applied perspective, the results support the use of MaxEnt for identifying potentially suitable areas, ecological boundaries, and zones requiring additional monitoring, but not as a substitute for field‐based abundance estimates. The most robust interpretation is achieved by combining SDMs with independent census data, camera‐trap monitoring, and other observation sources. Under a sharply continental climate, macroclimatic gradients define broad environmental potential, whereas realized density is shaped at finer spatial scales by resources, shelter, hydrology, anthropogenic transformation, and species‐specific spatial organization. This distinction between potential suitability and realized density is the central outcome of the comparison.

## 5 Conclusion

MaxEnt models for 14 species revealed spatially heterogeneous environmental potential, while the degree of correspondence between predicted habitat suitability and observed population density varied among species. The most robust positive relationships after spatial evaluation remained for wolf and roe deer, whereas accounting for spatial structure reduced statistical confidence for bobak marmot. Small samples for several species, differences in density‐survey methods, polygon‐level averaging of suitability, and the temporal mismatch between climatology and contemporary biological data limit cross‐species inference. MaxEnt is therefore useful for mapping potentially suitable areas but should not be used as a direct substitute for field estimates of abundance and density.

## Author Contributions

**Zarina Sergazinova:** conceptualization (equal), data curation (equal), formal analysis (equal), funding acquisition (equal), investigation (equal), methodology (equal), project administration (equal), resources (equal), software (equal), supervision (equal), validation (equal), visualization (equal), writing – original draft (equal), writing – review and editing (equal).

## Funding

This research was funded by the Science Committee of Science of the Ministry of Science and Higher Education of the Republic of Kazakhstan (Grant No. AP25795423 Geoinformation modeling and mapping of distribution and stock of hunting and commercial species of mammals of the northeast of Kazakhstan).

## Ethics Statement

The study employed non‐invasive census methods (departmental data from winter censuses and camera traps) that did not involve physical contact with the animals or disturb them; therefore, approval from an ethics committee was not required.

## Consent

The author has nothing to report.

## Conflicts of Interest

The author declares no conflicts of interest.

## Data Availability

The occurrence data used in this study include original field observations and publicly available records obtained from GBIF. The processed occurrence records used in the analyses are available through the following data repository: https://urldefense.com/v3/__https://drive.google.com/drive/folders/1UTaK7gTOv6khMFS5A37bWGX8‐T9p‐c72__;!!N11eV2iwtfs!oQGOg1Wmvne6qI1yOQbx0jPpnIoIZ0LRaqImOlKJxQB6clU_yOiTJrGSmSeKkZ0YOcFWGycFv4aRci02STENQyH9AfK8CHHb$. The camera‐trap dataset generated during this study is publicly available through GBIF at DOI: 10.15468/xknn3b.

## References

[ece374621-bib-0001] Afanasyev, A. V. , V. S. Bazhanov , M. N. Korelov , et al. 1953. Animals of Kazakhstan; Academy of Sciences of the Kazakh SSR Publishing House: Alma‐Ata, 536. Publishing House of the Academy of Sciences of the Kazakh SSR.

[ece374621-bib-0002] Ahmadpour, M. , S. Shokri , H. Varasteh‐Moradi , M. Waltert , I. Khorozyan , and M. Soofi . 2021. “The Occurrence of Rare Corsac Fox ( *Vulpes corsac* ) in Iran Is Mainly Determined by Prey Presence and Land Use.” Journal of Arid Environments 189: 104475. 10.1016/j.jaridenv.2021.104475.

[ece374621-bib-0003] Allen, A. W. , and R. D. Hoffman . 1984. “Habitat Suitability Index Models: Muskrat.” Accessed March 24, 2026. https://pubs.usgs.gov/publication/fwsobs82_10_46.

[ece374621-bib-0004] Allen, M. L. , K. C. B. Weiss , and A. M. Green . 2025. “Ecological and Anthropogenic Drivers of Red Fox ( *Vulpes vulpes* ) Abundance and Site Use Across the Contiguous USA.” Journal of Biogeography 52: e70008. 10.1111/jbi.70008.

[ece374621-bib-0005] Aryn, E. M. 2003. Pavlodar Irtysh Region: Encyclopedia, 676. Evero.

[ece374621-bib-0006] Bae, H.‐K. , J.‐K. Lee , T.‐K. Eom , D.‐H. Lee , and S.‐J. Rhim . 2021. “Ecological Factors Influencing the Selection of Sett Location by the Asian Badger *Meles leucurus* .” Wildlife Biology 2021: wlb.00910. 10.2981/wlb.00910.

[ece374621-bib-0007] Bazarbekov, K. U. , and O. V. Lyakhov . 2005. Wildlife of Pavlodar Irtysh Region (Vertebrates), 336. “ECO” Scientific Production Firm LLP.

[ece374621-bib-0008] Belov, V. N. 1931. “Review of Rodents of Northern Kazakhstan.” Proceedings. Plant Protection in Siberia 1: 164–182.

[ece374621-bib-0009] Biancolini, D. , M. Pacifici , M. Falaschi , et al. 2024. “Global Distribution of Alien Mammals Under Climate Change.” Global Change Biology 30: e17560. 10.1111/gcb.17560. 39545282

[ece374621-bib-0010] CGIAR‐CSI SRTM . 2026. Accessed March 24, 2026. http://srtm.csi.cgiar.org/.

[ece374621-bib-0011] Dupal, T. A. , Z. M. Sergazinova , N. T. Erzhanov , and Y. N. Litvinov . 2017. “Preliminary Assessment of Changes in the Structure of Small Mammal Communities Caused by Industrial Pollution in the North Kazakhstan Region.” Contemporary Problems of Ecology 10: 700–706. 10.1134/S199542551706004X.

[ece374621-bib-0012] Dussault, C. , J. P. Ouellet , R. Courtois , J. Huot , L. Breton , and J. Larochelle . 2004. “Behavioural Responses of Moose to Thermal Conditions in the Boreal Forest.” Ecoscience 11: 321–328. 10.1080/11956860.2004.11682839.

[ece374621-bib-0013] Elith, J. , C. H. Graham , R. P. Anderson , et al. 2006. “Novel Methods Improve Prediction of Species' Distributions From Occurrence Data.” Ecography 29: 129–151. 10.1111/j.2006.0906-7590.04596.x.

[ece374621-bib-0014] Elith, J. , and J. R. Leathwick . 2009. “Species Distribution Models: Ecological Explanation and Prediction Across Space and Time.” Annual Review of Ecology, Evolution, and Systematics 40: 677–697. 10.1146/annurev.ecolsys.110308.120159.

[ece374621-bib-0015] Elith, J. , S. J. Phillips , T. Hastie , M. Dudík , Y. E. Chee , and C. J. Yates . 2011. “A Statistical Explanation of MaxEnt for Ecologists.” Diversity and Distributions 17: 43–57. 10.1111/j.1472-4642.2010.00725.x.

[ece374621-bib-0016] Eom, T.‐K. , J.‐K. Lee , D.‐H. Lee , H. Ko , and S.‐J. Rhim . 2023. “Adaptive Response of Siberian Roe Deer *Capreolus pygargus* to Climate and Altitude in the Temperate Forests of South Korea.” Wildlife Biology 2023: e01138. 10.1002/wlb3.01138.

[ece374621-bib-0017] Erzhanov, N. T. , V. A. Kamkin , U. V. Ubaskin , et al. 2016. Biodiversity of Pavlodar Region, 496. S. Toraighyrov Pavlodar State University.

[ece374621-bib-0018] Eshtiaghi, A. , S. Naderi , A. Mohammadi , and H. Y. Wan . 2024. “Identifying Wild Boar ( *Sus scrofa* ) Crop Damage Hotspots to Mitigate Human‐Wild Boar Conflicts in Northern Iran.” Global Ecology and Conservation 54: e03065. 10.1016/j.gecco.2024.e03065.

[ece374621-bib-0019] Ferreiro‐Arias, I. , E. García , V. Palacios , et al. 2024. “Drivers of Wolf Activity in a Human‐Dominated Landscape and Its Individual Variability Toward Anthropogenic Disturbance.” Ecology and Evolution 14: e70397. 10.1002/ece3.70397. PMC11494153 39440215

[ece374621-bib-0020] Filla, M. , J. Premier , N. Magg , et al. 2017. “Habitat Selection by Eurasian lynx ( *Lynx lynx* ) is Primarily Driven by Avoidance of Human Activity During Day and Prey Availability During Night.” Ecology and Evolution 7: 6367–6381. 10.1002/ece3.3204. PMC5574813 28861240

[ece374621-bib-0021] GBIF.org . 2026a. “ *Meles leucurus* Occurrence Download.” Accessed February 25, 2026. 10.15468/dl.yhbnw4.

[ece374621-bib-0022] GBIF.org . 2026b. “ *Sciurus vulgaris* Occurrence Download.” Accessed February 25, 2026. 10.15468/dl.6jp8fw.

[ece374621-bib-0023] GBIF.org . 2026c. “ *Canis lupus* Occurrence Download.” Accessed February 25, 2026. 10.15468/dl.yvwv58.

[ece374621-bib-0024] GBIF.org . 2026d. “ *Mustela erminea* Occurrence Download.” Accessed February 25, 2026. 10.15468/dl.fjtez8.

[ece374621-bib-0025] GBIF.org . 2026e. “ *Lepus timidus* Occurrence Download.” Accessed February 25, 2026. 10.15468/dl.suwyv3.

[ece374621-bib-0026] GBIF.org . 2026f. “ *Sus scrofa* Occurrence Download.” Accessed February 25, 2026. 10.15468/dl.49vbp9.

[ece374621-bib-0027] GBIF.org . 2026g. “ *Alces alces* Occurrence Download.” Accessed February 25, 2026. 10.15468/dl.b7t7wa.

[ece374621-bib-0028] GBIF.org . 2026h. “ *Ondatra zibethicus* Occurrence Download.” Accessed February 25, 2026. 10.15468/dl.r7sve9.

[ece374621-bib-0029] GBIF.org . 2026i. “ *Marmota bobak* Occurrence Download.” Accessed February 25, 2026. 10.15468/dl.7sv9xa.

[ece374621-bib-0030] GBIF.org . 2026j. “ *Capreolus pygargus* Occurrence Download.” Accessed February 25, 2026. 10.15468/dl.sxe5g9.

[ece374621-bib-0031] GBIF.org . 2026k. “ *Vulpes corsac* Occurrence Download.” Accessed March 2, 2026. 10.15468/dl.zqur9b.

[ece374621-bib-0032] GBIF.org . 2026l. “ *Mustela eversmanii* Occurrence Download.” Accessed March 2, 2026. 10.15468/dl.fe9sw2.

[ece374621-bib-0033] GBIF.org . 2026m. “ *Lynx lynx* Occurrence Download.” Accessed March 11, 2026. 10.15468/dl.py5b8x.

[ece374621-bib-0034] GBIF.org . 2026n. “ *Vulpes vulpes* Occurrence Download.” Accessed March 11, 2026. 10.15468/dl.kfe2ng.

[ece374621-bib-0035] Göransson, A. , D. Carniato , L. Hillstrom , P. Hillborg , M. Larsson , and A. J. Carpio . 2025. “The Influence of Snow Cover, Elevation, and Latitude on Fur Colour in the Mountain Hare.” Ecosistemas: Revista Cietifica y Tecnica de Ecologia y Medio Ambiente 34: 2856. 10.7818/ECOS.2856.

[ece374621-bib-0036] Granata, M. , F. Di Paolo , A. M. Belfiore , et al. 2026. “Habitat Suitability and Niche Comparison of Sympatric Weasels in Northwestern Italy.” Mammal Research 71: 8. 10.1007/s13364-025-00831-z.

[ece374621-bib-0037] Greenberg, S. , T. Godin , and J. Whittington . 2019. “Design Patterns for Wildlife‐Related Camera Trap Image Analysis.” Ecology and Evolution 9, no. 24: 13706–13730. 10.1002/ece3.5767. PMC6953665 31938476

[ece374621-bib-0038] Insebayev, T. A. , A. D. Azerbayev , A. D. Abdrakhmanova , I. V. Randyuk , and Z. K. Shengenova . 2007. Pavlodar Region: Pages of History (1938–2008). Vol. Part 1, 330. S. Toraighyrov Pavlodar State University.

[ece374621-bib-0039] Kiseleva, N. V. 2024. “Depression of the Population of the Steppe Polecat ( *Mustela eversmanii* ) in the Southern Urals.” Biological Bulletin 2: 276–285. 10.31857/S1026347024020105.

[ece374621-bib-0040] Korpela, K. , P. Helle , H. Henttonen , et al. 2014. “Predator‐Vole Interactions in Northern Europe: The Role of Small Mustelids Revised.” Proceedings of the Royal Society B 281: 20142119. 10.1098/rspb.2014.2119. PMC4241000 25355481

[ece374621-bib-0041] Koshkina, A. , I. Grigoryeva , V. Tokarsky , et al. 2020. “Marmots From Space: Assessing Population Size and Habitat Use of a Burrowing Mammal Using Publicly Available Satellite Images.” Remote Sensing in Ecology and Conservation 6: 153–167. 10.1002/rse2.138.

[ece374621-bib-0042] Kudrenko, S. , V. Fenchuk , J. Vollering , et al. 2023. “Walking on the Dark Side: Anthropogenic Factors Limit Suitable Habitat for Gray Wolf ( *Canis lupus* ) in a Large Natural Area Covering Belarus and Ukraine.” Global Ecology and Conservation 46: e02586. 10.1016/j.gecco.2023.e02586.

[ece374621-bib-0043] Kuznetsov, B. A. 1948. Mammals of Kazakhstan; MOIP Publishing House, 226. Moscow Society of Naturalists (MOIP).

[ece374621-bib-0044] Lawlor, J. , L. Comte , G. Grenouillet , et al. 2024. “Mechanisms, Detection and Impacts of Species Redistributions Under Climate Change.” Nature Reviews Earth and Environment 5: 351–368. 10.1038/s43017-024-00527-z.

[ece374621-bib-0045] Lee, O. , P. E. Schlichting , and Y.‐S. Jo . 2022. “Habitat Model for Wild Boar ( *Sus scrofa* ) in Bukhansan National Park, Seoul.” Journal of Urban Ecology 8: juac027. 10.1093/jue/juac027.

[ece374621-bib-0046] Lenarz, M. , M. Nelson , M. Schrage , and A. Edwards . 2009. “Temperature Mediated Moose Survival in Northeastern Minnesota.” Journal of Wildlife Management 73: 503–510. 10.2193/2008-265.

[ece374621-bib-0047] Li, J. , D. Li , Y. Fu , Y. Ma , J. Ge , and L. Feng . 2024. “The Abundance and Sett Ecological Characteristics of the Asian Badger ( *Meles leucurus* ) in the Northeast Tiger and Leopard National Park (NE China).” Global Ecology and Conservation 56: e03272. 10.1016/j.gecco.2024.e03272.

[ece374621-bib-0048] Li, X. , Z. Jiang , C. Li , S. Tang , F. Li , and C. Li . 2020. “Climate and Environmental Determinants of Mammal Distribution Patterns in Central Asia.” Global Ecology and Conservation 24: e01330. 10.1016/j.gecco.2020.e01330.

[ece374621-bib-0049] Lim, S. , M. Banjade , S. Park , E.‐K. Kim , and Y. Park . 2025. “Proximity to Water Sources and Avoidance of Human Settlements Drive Wild Boar ( *Sus scrofa* ) Occupancy During Spring in an Urban‐Proximate National Park in South Korea.” Animals 15: 3423. 10.3390/ani15233423. PMC12691439 41375481

[ece374621-bib-0050] Litvinov, Y. N. , T. A. Dupal , N. T. Erzhanov , et al. 2015. “Aspects of Shrew Community Organization in Open Landscapes of Siberia and Northern Kazakhstan.” Contemporary Problems of Ecology 2: 259–267. 10.1134/s1995425515020110.

[ece374621-bib-0051] Mancini, G. , M. Di Marco , M. Carboni , P. Cerretti , L. Maiorano , and L. Santini . 2025. “On the Importance of Expert‐Informed Variable Selection in Species Distribution Modelling.” Journal of Biogeography 52: e70037. 10.1111/jbi.70037.

[ece374621-bib-0052] Marin, C. , J. Werno , G. Le Campion , and L. Couderchet . 2024. “Navigating Discreetly: Spatial Ecology of Urban Wild Boar in Bordeaux City's Landscape of Fear, France.” Science of the Total Environment 954: 176436. 10.1016/j.scitotenv.2024.176436. 39312969

[ece374621-bib-0053] Martino, G. , G. Chiatante , M. Ferloni , and A. Meriggi . 2024. “Population Trend and Distribution of Mountain Hare ( *Lepus timidus* ) and Brown Hare ( *Lepus europaeus* ) in the Central Alps (Northern Italy, 1980–2020).” European Journal of Wildlife Research 70: 38. 10.1007/s10344-024-01791-6.

[ece374621-bib-0054] Melvin, G. P. , and J. Bowman . 2026. “Marsh Interspersion and Muskrat ( *Ondatra zibethicus* ) Habitat Use.” Ecology and Evolution 16: e73155. 10.1002/ece3.73155. PMC12952997 41783353

[ece374621-bib-0055] Merow, C. , M. J. Smith , and J. A. Silander . 2013. “A Practical Guide to MaxEnt for Modeling Species' Distributions: What It Does, and Why Inputs and Settings Matter.” Ecography 36: 1058–1069. 10.1111/j.1600-0587.2013.07872.x.

[ece374621-bib-0056] Murray, D. , E. Cox , W. Ballard , et al. 2006. “Pathogens, Nutritional Deficiency, and Climate Influences on a Declining Moose Population.” Wildlife Monographs 166: 1–30. 10.2193/0084-0173(2006)166[1:PNDACI]2.0.CO;2.

[ece374621-bib-0057] Pavlodar Society of Hunters and Fishermen . n.d. “Hunting Grounds Map.” Accessed February 25, 2026. https://ohota‐pavlodar.kz/ssl/u/3e/336bb2013011ed8988c832f0035ee6/‐/%D0%BA%D0%B0%D1%80%D1%82%D0%B0%20%D0%BD%D0%BE%D0%B2%D0%B0%D1%8F%2005.11.jpg.

[ece374621-bib-0058] Peterson, A. T. , J. Soberón , R. G. Pearson , et al. 2011. Ecological Niches and Geographic Distributions. Princeton University Press. 10.1515/9781400840670.

[ece374621-bib-0059] Phillips, S. J. , R. P. Anderson , and R. E. Schapire . 2006. “Maximum Entropy Modeling of Species Geographic Distributions.” Ecological Modelling 190: 231–259. 10.1016/j.ecolmodel.2005.03.026.

[ece374621-bib-0060] Reshamwala, H. S. , A. Bhattacharya , S. Khan , et al. 2022. “Modeling Potential Impacts of Climate Change on the Distribution of Wooly Wolf ( *Canis lupus chanco* ).” Frontiers in Ecology and Evolution 10: 815621. 10.3389/fevo.2022.815621.

[ece374621-bib-0061] Reyserhove, L. , B. Norton , and P. Desmet . 2023. Best Practices for Managing and Publishing Camera Trap Data. GBIF Secretariat. 10.35035/doc-0qzp-2x37.

[ece374621-bib-0062] Ripari, L. , J. Premier , E. Belotti , et al. 2022. “Human Disturbance Is the Most Limiting Factor Driving Habitat Selection of a Large Carnivore Throughout Continental Europe.” Biological Conservation 266: 109446. 10.1016/j.biocon.2021.109446.

[ece374621-bib-0063] Ruco, V. , and F. Marucco . 2025. “A Game of Risk: Human Activities Shape Roe Deer Spatial Behavior in Presence of Wolves in the Southwestern Alps.” Diversity 17: 115. 10.3390/d17020115.

[ece374621-bib-0064] Sergazinova, Z. 2026. Terrestrial Mammal Camera‐Trap Monitoring in Forest and Floodplain Ecosystems of Northeastern Kazakhstan [Data set]. Toraighyrov University. GBIF.org. 10.15468/xknn3b.

[ece374621-bib-0065] Sergazinova, Z. M. , A. V. Makarov , and N. T. Erzhanov . 2025. “Influence of Anthropogenic Pollution on Ecological and Morphological Characteristics of the Narrow‐Headed Vole (Stenocranius Gregalis Pallas, 1779) in Steppe Ecosystems of the Pavlodar Pre‐Irtysh Region.” Journal of Ecology and Environment 49: 14. 10.5141/jee.25.023.

[ece374621-bib-0066] Sergazinova, Z. M. , A. V. Makarov , A. T. Toleuzhanova , A. N. Kukusheva , S. Z. Arynova , and I. Y. Chidunchi . 2025. “Comparative Characterization of Small Mammal Communities in Forest‐Steppe and Steppe Landscapes of the South of Western and Central Siberia.” OnLine Journal of Biological Sciences 25: 150–162. 10.3844/ojbsci.2025.150.162.

[ece374621-bib-0067] Shnitnikov, V. N. 1935. Part 2. “Northern Kazakhstan.” In The Animal World of Kazakhstan, 242. Kazakhstanskoe Kraevoe Izdatelstvo.

[ece374621-bib-0068] Sludsky, A. A. , ed. 1977. Mammals of Kazakhstan, in 4 Vols. Vol. 1, Part 2, 536. Nauka KazSSR Publishing House.

[ece374621-bib-0069] Sludsky, A. A. , ed. 1978. Mammals of Kazakhstan, in 4 Vols. Vol. 1, Part 3, 492. Nauka KazSSR Publishing House.

[ece374621-bib-0070] Sludsky, A. A. , ed. 1985. Mammals of Kazakhstan, in 4 Vols. Vol. 4, 279. Nauka KazSSR Publishing House.

[ece374621-bib-0071] Suárez‐Tangil, B. D. , and A. Rodríguez . 2021. “Uniform Performance of Mammal Detection Methods Under Contrasting Environmental Conditions in Mediterranean Landscapes.” Ecosphere 12, no. 3: e03349. 10.1002/ecs2.3349.

[ece374621-bib-0072] Teitelbaum, C. S. , A. P. K. Sirén , E. Coffel , et al. 2021. “Habitat Use as Indicator of Adaptive Capacity to Climate Change.” Diversity and Distributions 27: 655–667. 10.1111/ddi.13223.

[ece374621-bib-0073] USGS ESPA . 2026. “Earth Resources Observation and Science Center Science Processing Architecture.” Accessed March 24, 2026. https://espa.cr.usgs.gov/index/.

[ece374621-bib-0074] van Beest, F. M. , B. Van Moorter , and J. M. Milner . 2012. “Temperature‐Mediated Habitat Use and Selection by a Heat‐Sensitive Northern Ungulate.” Animal Behaviour 84: 723–735. 10.1016/j.anbehav.2012.06.032.

[ece374621-bib-0075] van den Bosch, M. , K. F. Kellner , M. G. Gantchoff , et al. 2023. “Habitat Selection of Resident and Non‐Resident Gray Wolves: Implications for Habitat Connectivity.” Scientific Reports 13: 20415. 10.1038/s41598-023-47815-0. PMC10663587 37990118

[ece374621-bib-0076] WorldClim . 2026. Accessed March 24, 2026. https://worldclim.org/.

[ece374621-bib-0077] Zhang, W. , X. Liu , T. Zhang , and G. Cui . 2025. “Integrating a Newcomer: Niche Differentiation and Habitat Use of Eurasian Red Squirrels and Native Species in a Forest Reserve Under Human Disturbance.” Forests 16: 1360. 10.3390/f16081360.

[ece374621-bib-0078] Zorzi, P. , A. Nardotto , M. Bottazzo , and M. Dal Zotto . 2022. “Habitat Selection of the Roe Deer *Capreolus capreolus* (Artiodactyla, Cervidae) in an Agroforestry System.” Natural History Sciences 9: 3–6. Accessed March 24, 2026. https://sisn.pagepress.org/nhs/article/view/550. 10.4081/nhs.2022.550.

